# Strategies for developing sustainable communities in higher education institutions

**DOI:** 10.1038/s41598-023-48021-8

**Published:** 2023-11-23

**Authors:** Alberto Biancardi, Annarita Colasante, Idiano D’Adamo, Cinzia Daraio, Massimo Gastaldi, Antonio Felice Uricchio

**Affiliations:** 1 Department Studies, Monitoring and International Relations, Gestore dei Servizi Energetici GSE S.P.A., Rome, Italy; 2 Department of Law and Economics, UnitelmaSapienza University of Rome, Rome, Italy; 3 Department of Computer, Control and Management Engineering, Sapienza University of Rome, Rome, Italy; 4 Department of Industrial and Information Engineering and Economics, University of L’Aquila, L’Aquila, Italy; 5 President of the Governmental Agency for the Evaluation of Universities and Research System (ANVUR), Rome, Italy

**Keywords:** Environmental social sciences, Energy and society

## Abstract

Higher education institutions (HEIs), based on learning, innovation, and research, can support the progress of civil society. Many HEIs are implementing sustainability practices and projects to counteract climate change, often involving youth participation. The present study aimed at identifying how sustainable communities may be fostered in a university setting. To that end, a questionnaire was administered to engineering students at the start and end of a course on energy issues, assessing their perceptions of sustainability using multi-criteria decision analysis. The results showed that students placed greater value on sustainability at the end of the course. Additionally, the findings highlight that the implementation of projects aimed at tackling real problems may be useful for disseminating knowledge and sustainable practices. The main implications of this study indicate that sustainable communities in academia lay on six foundational pillars: sustainable education, energy (and resource) independence, subsidies in support of the green economy, initiatives aimed at reducing the carbon footprint, energy community development, and new green professional opportunities.

## Introduction

The historical disregard for ecosystem balance has led to escalating temperatures and catastrophic climate events, with adverse impacts on both humanity and the environment. Consequently, sustainability has become an integral component of scientific discourse and government agendas^1,2^. Nonetheless, significant disparities exist on regional and global scales, concerning the adoption and advocacy of sustainable practices^3^. The 1987 Brundtland Report defined sustainability as that concept in which the needs of current generations can be met without compromising the needs of future generations^4^. The Sustainable Development Goals (SDGs), comprising part of the broader 2030 Agenda, outline pragmatic and impartial strategies for addressing global challenges while benefitting the majority of stakeholders^5^. Companies are called upon to adopt approaches based on corporate social responsibility(CSR)^6^, with a goal of reducing the level of pollutant emissions^7^. Similarly, the concepts of integration between city and industry need to be reviewed, and the need to create green spaces arises^8^. Importantly, the literature also emphasizes that sustainability aims at promoting the needs of future generations and discouraging selfish behaviour in the present^9^. Similarly, it is crucial to provide sustainability analyses that are able to bring different points of view together^10,11^.

The European Green Deal is seen as a growth strategy with which the Europe is to become a fair and prosperous society with a modern, resource-efficient and competitive economy. It includes proposals for measures to reduce emissions in various areas, such as agriculture, mobility, building renovation, sustainable financing, energy systems or research and development. Some authors underline that a “generative approach” to economy can represent the “social vaccine” in order to be resilient to current and future pandemics. It is crucial to focus on active and healthy ageing and on combating NEET phenomenon (young people who neither work nor study)^12^.

Higher education institutions (HEIs) can support the achievement of the SDGs^13^ and, in particular, SDG 4 (i.e., quality education)^14^. As Pope Francesco stressed, we need “a new kind of education, one that allows us to overcome the current globalization of indifference and culture of waste.” In this direction, the phenomenon of “sustainability washing” should be avoided, and the management of sustainability courses and the sustainable adaptation of educational curricula should be entrusted to those with relevant experience. The knowledge triangle, encompassing education, innovation, and research, must collaboratively support the progress of civil society^15^.

HEIs often possess strong localized affiliations that can serve as catalysts for the socioeconomic advancement of local ecosystems^16^. Concurrently, models of competitiveness drive internationalization^17^ and efficiency^18^, fostering tighter synergies between universities and urban environments capable of addressing the challenges of sustainable development^19^. To facilitate these endeavors, a framework of cooperative and virtuous human behavior is needed^20,21^.

The core mission of many HEIs is to ensure high employability for graduates. However, HEIs also acknowledge the profound significance of the SDGs^22^. Despite this, many sustainability topics remain inadequately covered within curricula^23^. Instead, HEIs predominantly address sustainability through campus operations and institutional initiatives^24^. HEIs recognize that students hold a decisive role in advancing sustainability initiatives^25^, within a tetrahedron structure. In this structure, students are positioned at the center, and the vertices are represented by alliances, professors, student competencies, and teaching methodology^26^. Some authors have highlighted the importance of understanding how specific pedagogical approaches may support the development of particular competencies^27^. Notably, research has shown that certain courses can heighten students’ interest in pursuing careers in the sustainability field^28^. Moreover, knowledge of sustainability issues may be useful for various professional activities that students may be called upon to address in their future careers^29^.

Student projects aimed at solving real problems have proven effective in kindling their engagement with sustainability concerns^30^. Such experiential projects may empower students to interface with the external world, honing their ability to assess multifaceted, complex issues^31^. This engagement may foster collaboration and encourage the pursuit of strategies that align with team requirements^32^. Thus, “living lab” models that put stakeholders at the center are strongly recommended^33^, as such models encourage students to form strategies that integrate interdisciplinary content^34^.

New curricula and government actions are required to address the challenges of the ecological transition^35^. Students from various disciplines—including engineering^28^—must be called upon to propose technical solutions, while concurrently acknowledging social dimensions. The skills required for engineers to deal with the changes that may result from pursuit of the SDGs are not only normative, strategic, and systemic, but also conceptual^36^, as the sustainability challenge requires engineers to address open, complex, and interdisciplinary issues^37^. Thus, new pedagogy is needed to support a holistic education^38^ drawing on active learning methods, including problem solving and simulations^39,40^. The challenges posed by sustainability imperatives demand a new approach to classical engineering, in order to solve and manage situations characterized by uncertainty, emergence, and incomplete knowledge^41^.

Several of the SDGs refer to energy issues^42^. Notably, SDG 7 (affordable and clean energy), SDG 11 (sustainable cities and communities), and SDG 12 (responsible consumption and production) highlight the prominence of energy issues in achieving sustainable development. Energy communities can aid in the pursuit of SDGs^43,44^. The concept of energy communities introduces a novel societal model for the ecological transition^45^, characterized by diverse business models^46^ and technological approaches^47^. Such communities strive for net-zero energy consumption^48^, thereby contributing to the advancement of a green economy. The significance of social and structural arrangements in determining the stability of energy communities has been acknowledged^49^. In fact, the ability to participate in an energy community can be influenced most by the family and social networks^50^. Consequently, university courses focusing on energy must center their curriculum on sustainability, in order to bridge the gap between the energy sector and the SDGs. While the factors that promote success in sustainable education vary across countries, development of a green culture has been shown to be consistently important^51^. To this end, policy actions must be identified, new businesses related to green sources must be developed, and new models of organization should be proposed, with citizens actively involved^52^.

In a previous study, a questionnaire was administered to students at the start and end of a university course. The results showed that, over the period of the course, students’ knowledge about sustainability increased. Additionally, sustainable education and confidence in youth competency were identified as fundamental pillars of future civil society^53^. Building upon these findings, the present research aimed at assessing how engineering students’ perceptions of sustainability issues changed following their completion of a course focused on energy topics. Based on the findings, we present recommendations for future university courses concentrated on sustainability. The research aimed at filling a gap in the literature by identifying the most significant factors for fostering sustainability communities within HEIs.

## Materials and methods

The methodological approach taken in the present study replicated that of Sovacool et al. ^54^ in the energy field, utilizing a behavioral methodology that drew insights from economics, engineering, and psychology. The use of questionnaires with university students is well-established in the literature^55^. The present study closely adhered to the design outlined in previous research^53^, seeking to assess time trends in the subject matter.

### Questionnaire development

In the first phase, a pre-established questionnaire^53^ was reviewed by five international academic experts (40% women and mainly European) with at least 10 years’ experience in sustainability issues. In the second phase, the feedback from these experts was merged with input from the research team (which included more than simply the course faculty, in order to control for bias). This resulted in some revisions. Specifically, two questions on energy independence (i.e., composed only of renewables or based on an energy mix) and several questions on the location of renewable plants were added. Additionally, the economic value ranges for energy were widened. The questionnaire had the limitation of being lengthy, due to the need for comparison with the prior research (conducted in the previous year). Finally, the second phase of the research culminated when the questionnaire was validated by both the experts and the research team. In the third phase, the questionnaire was administered to students on two occasions: once at the start of the course and again at the end of the course. Of note, during the intervening period, the initial data collected were neither analyzed nor discussed. Lastly, in the fourth phase, the working group analyzed the main results and shared them with the students, alongside the findings from the previous year. This facilitated a discussion that offered insight into the students’ responses. The research concluded during the first two examination sessions (which involved more than half of the enrolled students), providing a platform for further discussion and exploration of the results.

### Educational characteristics

The student questionnaire was administered to students enrolled in a master's degree program (predominantly in the field of Management Engineering) at Sapienza University of Rome. All students were registered in a course titled “Economics and Management of Energy Sources and Services”, which was an optional (non-compulsory) course with a strong focus on sustainability, comprising 60 lecture hours. At the culmination of the course, 99 students completed the questionnaire, representing five more than the number who completed it at the beginning of the course. Compared to the previous year, an additional 33 students completed the questionnaire (marking a 50% increase), highlighting the growth in student interest in topics related to energy and sustainability. Likely, this upswing was also compounded by positive feedback from students who attended the course in the prior year.

Beyond theoretical lectures, the course actively engaged stakeholders, with a special focus on younger individuals. To this end, students from the previous year presented their projects to the classroom during the initial phase of the course, in order to convey the course expectations and demonstrate their support for the concept of sustainable communities. These presentations also served to acknowledge and celebrate the quality of the projects presented in the previous year, which were strongly oriented towards problem solving. Additionally, the course involved the participation of several experts in the field, underlining the significance of robust collaboration between the university and external stakeholders.

### Methodologies

The present study sought to assess the impact of course participation on students’ attitudes and behaviors concerning sustainability. Specifically, the questionnaire aimed at gauging the “treatment effect” resulting from the information imparted during the course. In accordance with the transformative learning approach^56^, it was assumed that students’ prior knowledge would play only a marginal role.

The appendix presents all of the questionnaire items (a total of 46) and the corresponding student responses (mainly in the form of Likert scale responses)^57,58^. The questionnaire was sent to students electronically via the course’s online platform and completed using a Google form (start in February and end in May 2023, respectively). Students were provided a 5-day window to complete the questionnaire, and their anonymity was guaranteed. The results were discussed with students also during the examination sessions in June and July 2023.

To bolster the robustness of the results, a sustainability metric was constructed using multi-criteria decision analysis (MCDA), consistent with current practice in science education^59,60^. MCDA is perfectly suited for the assessment of sustainable development, as articulated by Munda^61^: “Multi-criteria evaluation supplies a powerful framework for the implementation of the incommensurability principle”. In essence, it fulfils the objectives of inter/multi-disciplinarity (with respect to the research team), participation (with respect to the local community), and transparency (as all criteria are presented in their original form). Thus, MCDA is an appropriate tool for assessing both micro and macro sustainability policies^61^.

Criteria were identified on the basis of the questionnaire responses, and equal weight was assigned to all criteria, since there was no reason to prioritize one criterion over another-consistent with the approach used for the SDGs^9^. In addition, statistical tests were conducted. Since we are able to reject the hypothesis of normal distribution for almost all variables (see Supplementary material for details), we deploy the Kruskal–Wallis test that is a non-parametric test that obviates the need for normality in the underlying distributions^62^. The Kruskal–Wallis test is one of the most powerful tests for testing the null hypothesis (H_0_)—that is, whether a number of independent groups come from the same population or form populations with the same median^63^. In the present study, we used the Kruskal–Wallis test to assess the equality of distribution across all levels of categorical values for certain groups (e.g., those used in the MCDA). Furthermore, the correlation matrix, which is both square and symmetrical, allowed us to explore the existence of linear relationships between the examined variables. The correlation coefficient measures the strength and direction of the relationship between two variables, within a range of −1 to 1. Both of these methodologies are extensively employed in the field of education science^64,65^.

## Results

The subsequent sections present the results from the questionnaire, categorized according to different subject areas. The MCDA results (i.e., sustainable index) between the beginning and end of the course, along with the related statistical analyses, are also provided.

### Sociodemographic data

The sample was mainly composed of students enrolled in the academic year 2022–2023 (74%). Students’ average age was 23.5 years (compared to 23.7 in the previous year) and the majority were male (66% vs. 64% in the previous year). Most came from central Italy (84% vs. 85% in the previous year) and lived in a household (79% vs. 82% in the previous year). The percentage of students who were concurrently employed dropped from 69 to 61% over the course duration. Thus, as the summer period approached, more students were likely to be seeking part-time or long-term employment.

### Perceptions and behaviors regarding sustainability

The concept of sustainability encompasses environmental, social, and economic dimensions. However, not all students recognized this multidimensionality. As in the previous year, students’ accuracy in responding to the questionnaire improved from the beginning to the end of the course. Interestingly, two students exclusively focused on the environmental and economic dimensions of sustainability, respectively. Unfortunately, due to the anonymous nature of the responses, the presentation and discussion of the results did not shed light on the identity or motivations of these students. Another critical aspect is that the questionnaire was also completed by students who did not regularly attend classroom lectures (as the attendance rate was only about 70%).

**Figure 1:**
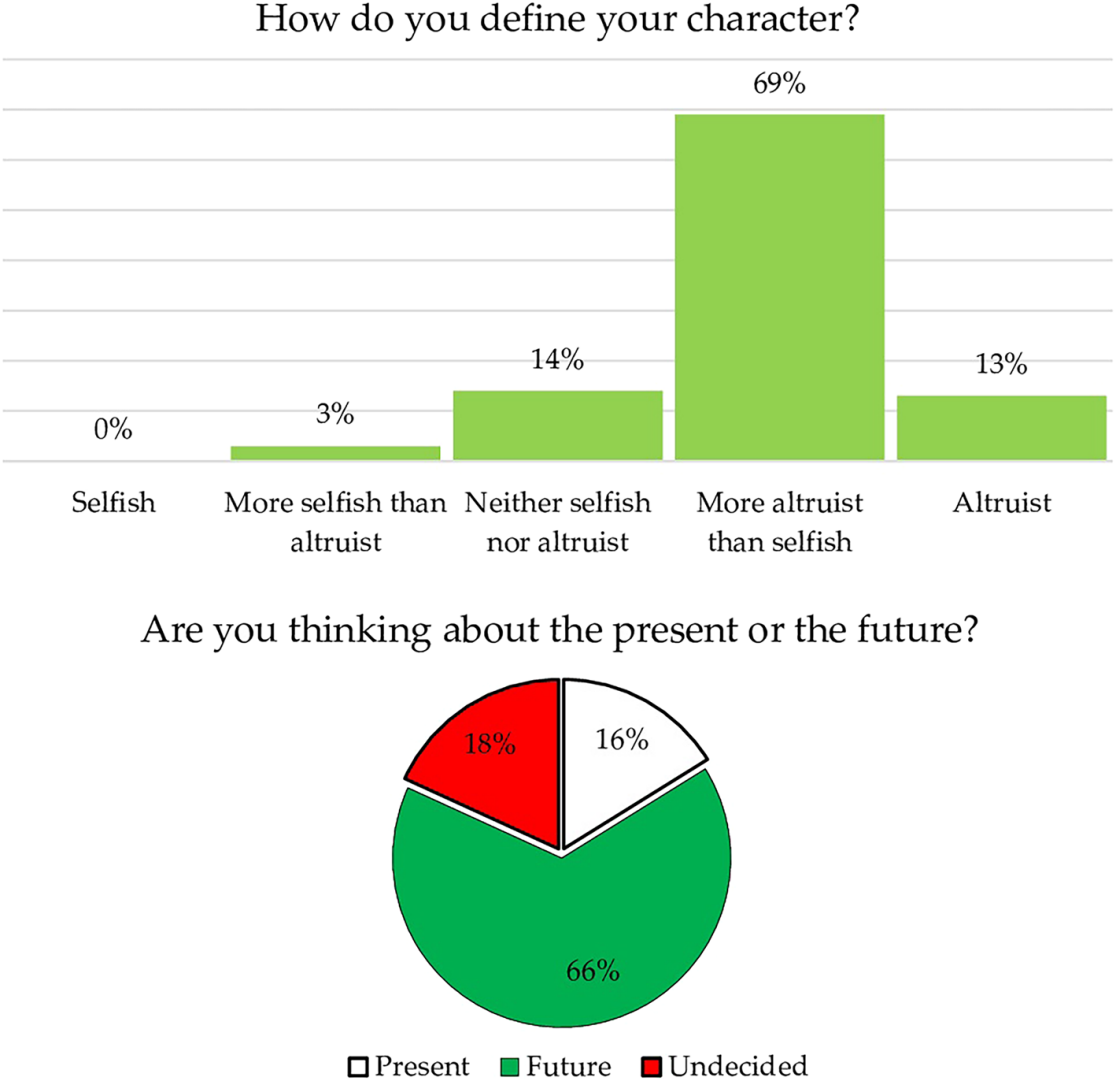
Degree of altruism and future-oriented perspective.

Students displayed a heightened inclination toward future considerations compared to the present (66% at the end of the course, up 4% from the beginning of the course and the previous year). They also tended to characterize themselves as more altruistic (scoring themselves as 3.9 on the 5-point scale) than selfish. A Mann–Whitney U test, which is akin to the Kruskal–Wallis test but used to compare only two groups, showed that students who were inclined toward the future (scoring 4.0) or undecided about the time horizon of their temporal perspective (scoring 3.9) tended to rate themselves as more altruistic than those leaning towards the present (scoring 3.3), who generally saw themselves as more neutral. Students emphasized a greater sense of responsibility as a factor influencing their focus on the future (Fig. 1).

**Figure 2:**
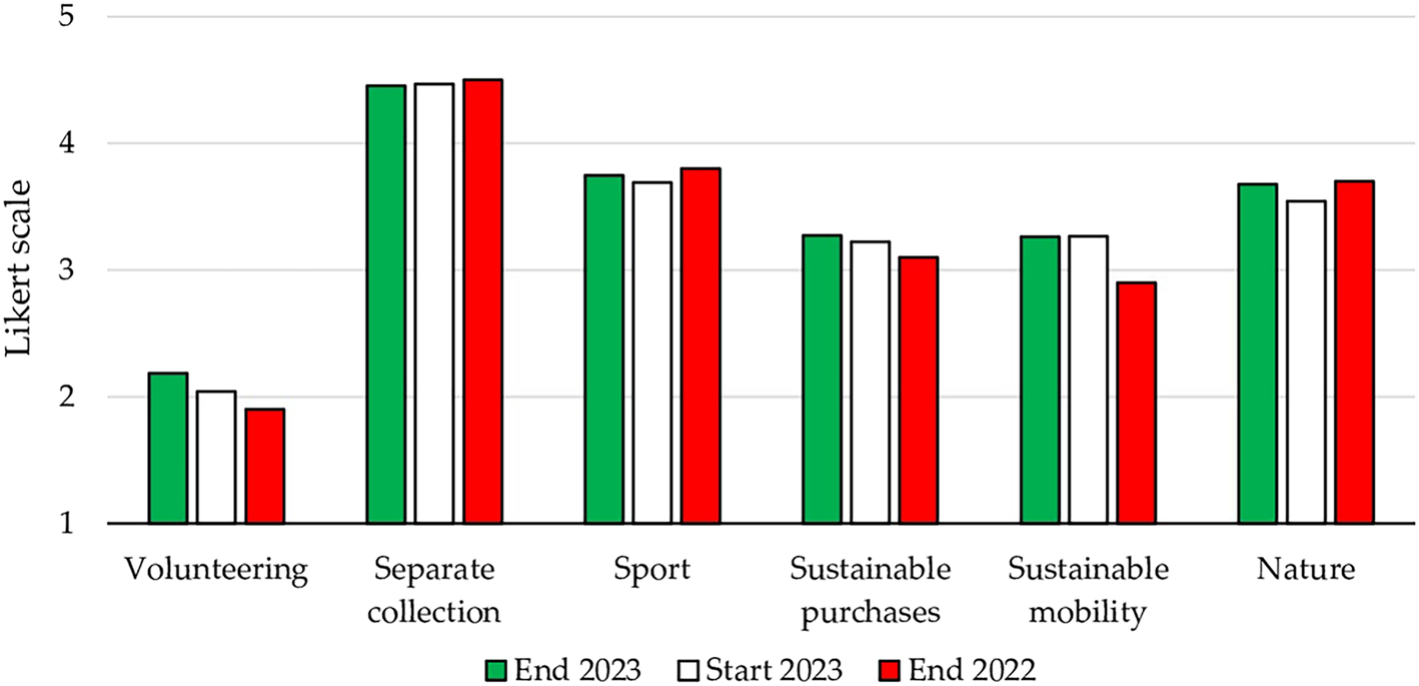
Mean values for sustainable behaviors: volunteering (2.2), separate collection (4.5), sport (3.7), sustainable shopping (3.3), sustainable mobility (3.3), nature (3.7) (1: never, 2: a few times, 3: sometimes, 4: often, 5: always).

With regard to sustainable behavior, no significant differences were observed from the beginning to the end of the course (+ 0.2 in nature and volunteerism), or in comparison to the previous year (+ 0.4 in sustainable mobility). Figure 2 illustrates the results for the relevant questions (see Supplementary material for the results of the Cronbach’s alpha). While separate waste collection was considered the most relevant, its implementation in Rome was perceived as inadequate. Consequently, there was a strong call for separate waste collection at home and at the university. Students also noted the inadequacy of university space for sports and nature activities. However, while they recognized the importance of sports and nature, they struggled to strike a balance between engaging in these activities and dedicating time to their studies. Sustainable mobility and sustainable purchasing also received notable emphasis. In support of the former, 48% of students reported walking 1 to 3 km daily and 38% reported walking 3 to 6 km daily. Regarding sustainable purchasing, students expressed an upper limit to the price they were willing to pay. During the discussion, it emerged that students associated sustainable products with the wealthiest consumers, and thereby the potential for social inequalities. Finally, the lower value assigned to volunteering was attributed to students’ time constraints, despite students expressing support for regular volunteer efforts.

### The role of energy policy

**Figure 3:**
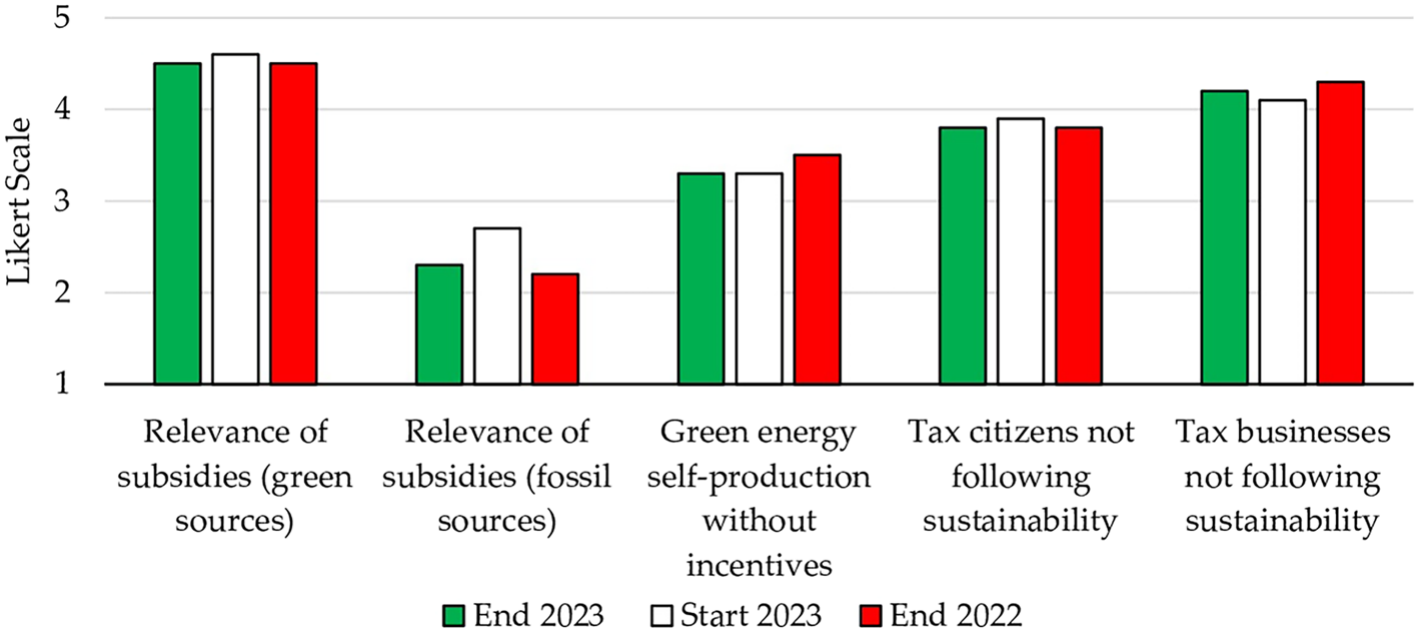
Items related to energy subsidies and taxes.

The advancement of renewable energy is closely linked with incentive policies. This is a well-justified policy approach, due to its positive externalities on both environmental and social fronts (Fig. 3)—see Supplementary material for the results of the Cronbach’s alpha.

Based on the Kruskal–Wallis test (*χ*^2^ = 13.57, *p* < 0.01), certain item mean rankings exhibited statistically significant differences. Consequently, the null hypothesis (indicating that the groups stemmed from the same population) was rejected. For the ensuing non-parametric pairwise multiple comparison procedure following the rejection of the Kruskal–Wallis test, Dunn’s test was adopted^66^. The post-hoc Dunn's test, utilizing a Bonferroni corrected alpha of 0.005, indicated a significant difference in the mean ranks between subsidies for green sources and subsidies for fossil fuels. At the beginning of the course, green subsidies held the highest rank (4.6), and at the end of the course, their rank was in line with the previous year (4.5). In general, the difference in values between the beginning and end of the course only concerned fossil fuel subsidies, which shifted from 2.7 to 2.3. We emphasized to students that this value did not denote sustainability, as it corresponded with the response “little agree”—a weaker stance than “not at all agree.” Students asserted that this still denoted a negative judgment on their part, perhaps influenced by certain government decrees that aimed at supporting citizens and businesses during the energy challenges arising from the conflict in Ukraine (e.g., by lowering costs for gasoline- or diesel-powered vehicles). The pronounced impact of government energy policies was reaffirmed by students’ neutral judgment about becoming a prosumer in the absence of incentives (3.3), which showed a decrease of 0.2 compared to the previous year. Likewise, students’ perception of potential taxes on behaviors aligning with sustainability principles persisted (remaining similar to the prior year). Students identified pollution as more attributable to businesses than citizens, substantiating their rationale for advocating for higher taxation for the former (4.2 for businesses and 3.8 for citizens).

### Willingness to pay for renewable sources

**Figure 4:**
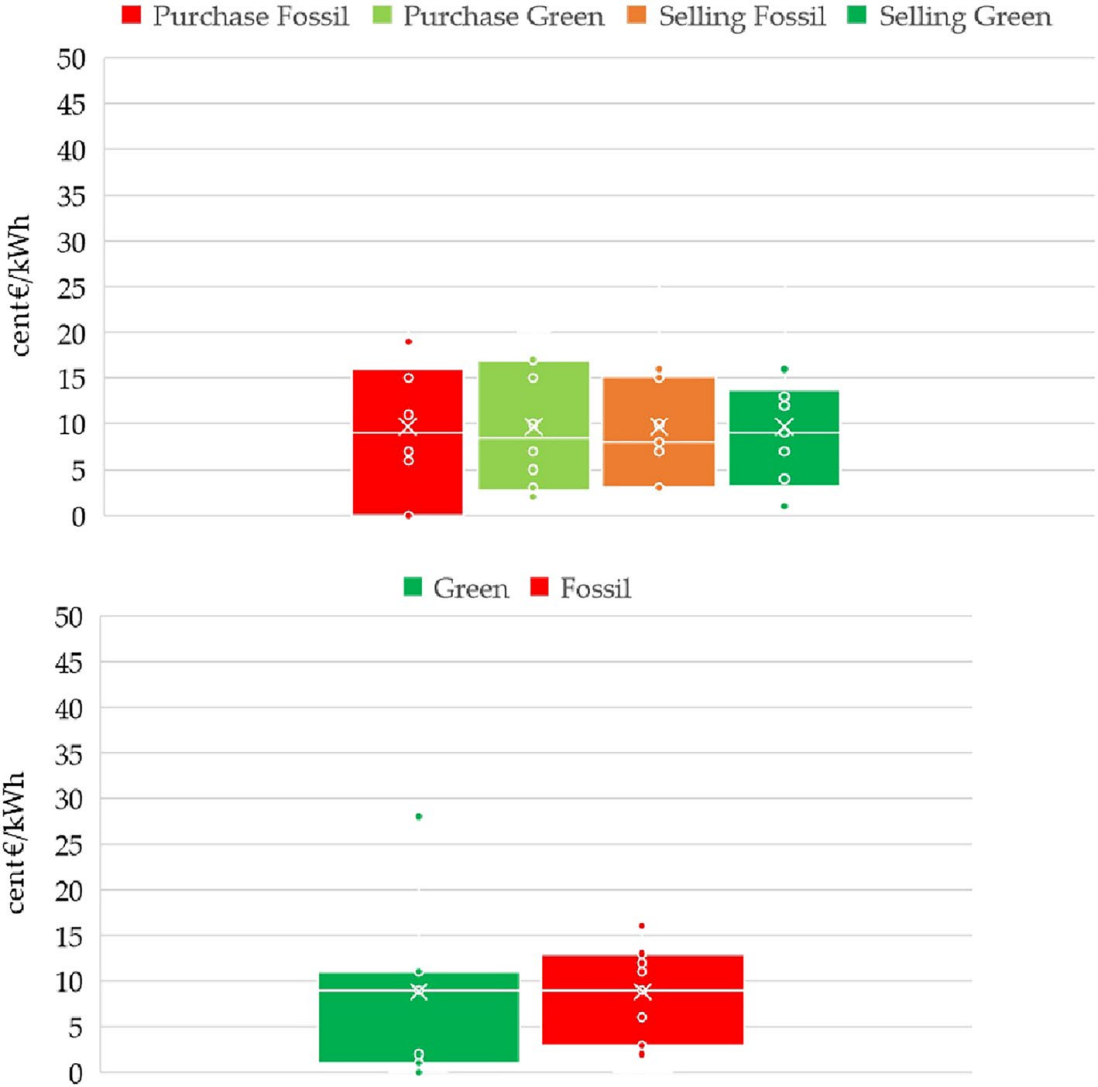
Average values for willingness to pay: 17.1 and 24.7 cent€/kWh for buying fossil fuels and green energy, respectively; and 19.5 and 23.8 cent€/kWh for selling fossil fuels and green energy, respectively. Average values for the bonus for energy produced and self-consumed: 7.3 and 2.6 cent€/kWh for green energy and fossil fuels, respectively.

A substantial portion of the questionnaire focused on economic dimensions, aimed at gauging students’ recognition of the value associated with different energy sources. The assessment encompassed a comparison between green energy and fossil fuels under two scenarios: one involving energy purchase and another involving energy sale. Furthermore, students were queried about their willingness to contribute to a subsidy for prosumer status (Fig. 4).

The results indicated that, in the context of energy purchase, students’ willingness to pay (WTP) for renewable sources at the end of the course was 7.6 cent€/kWh higher than that of fossil fuels. This difference in WTP exhibited a statistically significant increase from the beginning of the course (6.2 cent€/kWh) and from the previous year (6.6 cent€/kWh). Similarly, on the energy sale side, a similar trend emerged. The observed difference in WTP of 4.2 cent€/kWh in favor of renewable energy compared to fossil fuels exhibited an increase of 1.4 cent€/kWh compared to the start of the course and 2.0 cent€/kWh compared to the previous year.

The increase in monetary value could potentially be attributed to energy price inflation. Nonetheless, the data reveal an interesting pattern, whereby students did not perceive a significant difference between the selling and buying prices, possibly suggesting a common valuation assigned to green energy. Another interesting finding is the higher selling price associated with fossil fuels, compared to the purchase price. Students may have believed that consumers would be more inclined towards renewable sources, particularly given the higher price fetched from fossil fuels. This speculation could indicate that students perceived that sustainability could also be advanced by selling fossil fuels at an elevated price.

Further analysis of the subsidy for prosumer status reinforced the previous findings, showing elevated values compared to those recorded in the previous year (+ 2.7 and + 0.8 cent€/kWh for green and fossil fuels, respectively). This indicates a prevailing sentiment that subsidies should be in place for self-produced and consumed energy, irrespective of its environmental contribution. Such an attitude is likely influenced by existing policies, not only in the Italian system, but also in other contexts that provide subsidies for self-generated and self-consumed energy. However, it is important to emphasize that student opinions regarding subsidies for renewable sources remained unchanged over the course duration. In contrast, their valuation of a subsidy for being a fossil fuel prosumer decreased, resulting in a final value of 4.1 cent€/kWh.

### Greenwashing and the impact of the internet on sustainability

**Figure 5:**
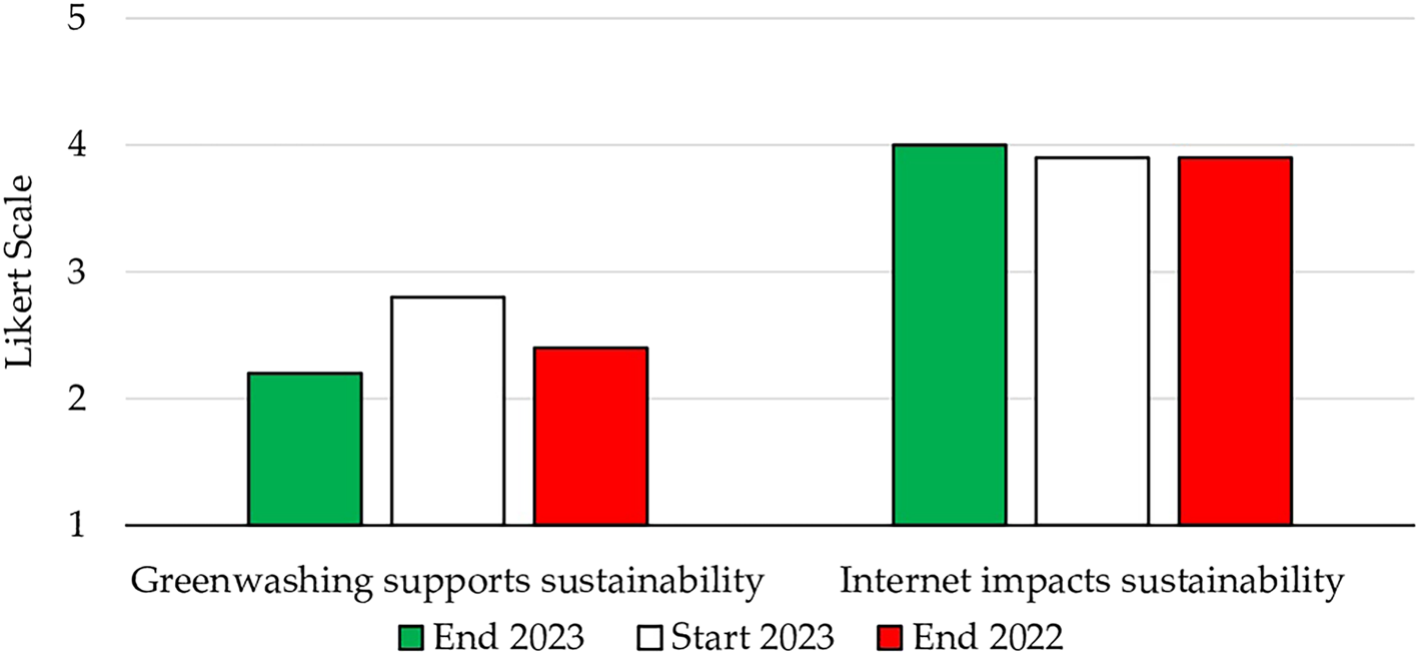
Greenwashing, the internet, and sustainability.

Sustainability includes not only finding solutions, but also avoiding insincere sustainability claims without genuine change. During the COVID-19 pandemic, the internet emerged as a crucial facilitator of sustained educational activities, reducing the demand for transportation. Furthermore, it underscored the potential for other activities to be carried out electronically (Fig. 5).

The results indicated a noteworthy enhancement in greenwashing, which showed the most significant reduction (-0.6) between the beginning and end of the course (on par with the previous year). However, the final rating of 2.2 suggests that students remained positioned within the realm of “little agree,” rather than “not at all agree,” with respect to the contribution of greenwashing to sustainable development. Some students who opted for this rating believed that greenwashing draws attention to the importance of sustainability. They argued that some businesses claim to follow CSR principles in order to maintain competitive advantage, even if they do not genuinely implement those principles. Other students connected greenwashing to the current culture of attention seeking (even if that attention is negative), driven by the dominance of social platforms. However, during the discussion of the results, several students argued that greenwashing contradicts the core principles of sustainability, as it propagates falsehoods.

Turning to the role of the internet, there was a clear convergence between the final rating (4.0) and the rating recorded at the beginning of the course and in the previous year (3.9). Evidently, the internet plays a vital role in fostering globalization. However, students emphasized that its extensive use should be moderated according to necessity. Acknowledging the internet’s profound impact on sustainability, students identified numerous job prospects aligned with digitization and sustainability. Consequently, they advocated for harmonious coexistence of these aspects.

### Sustainable education, professional opportunities, and the role of future generations

**Figure 6:**
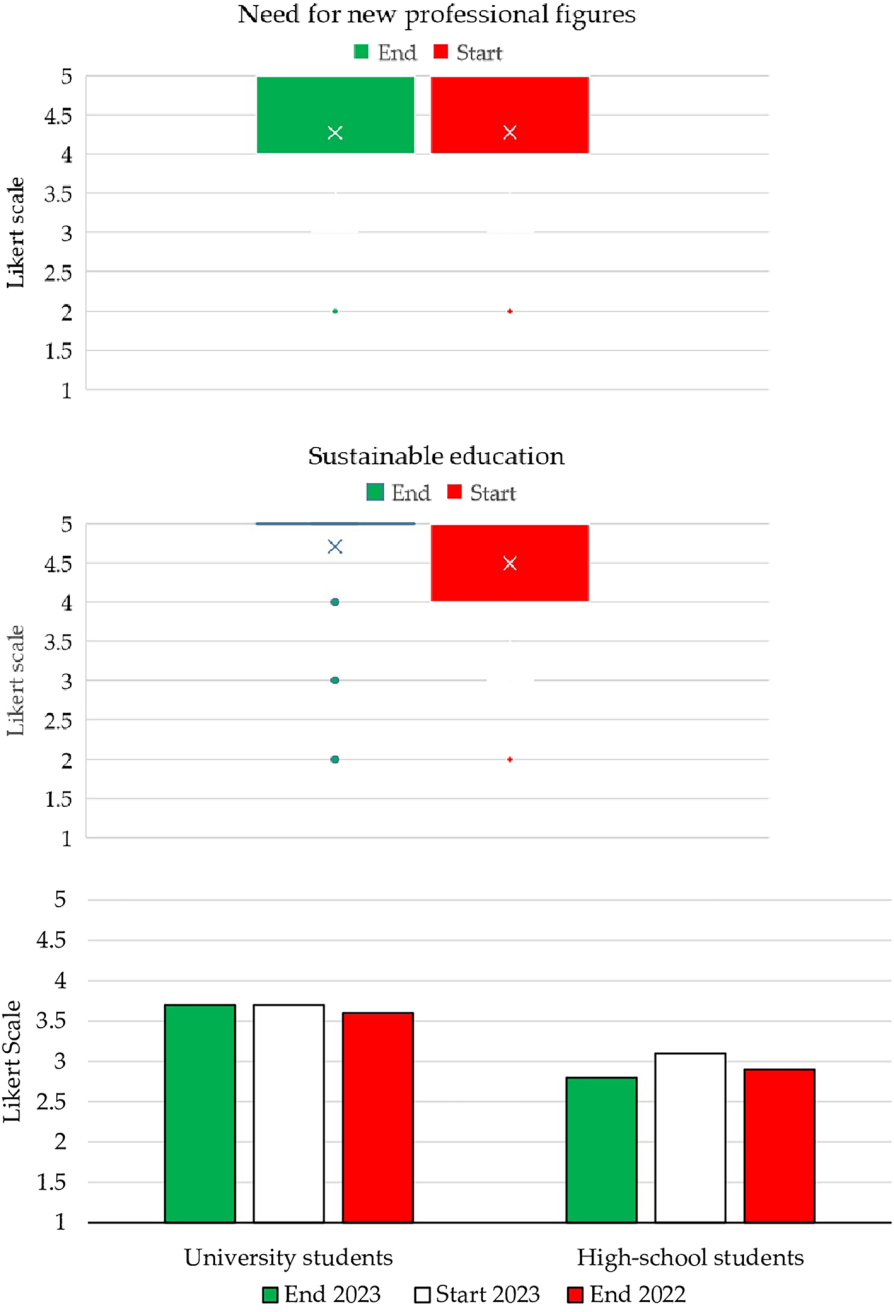
New professionals, sustainable education, and the role of future generations.

Students underscored the promising professional opportunities tied to sustainability, although their rating for this aspect slightly dipped compared to the previous year (4.3 vs. 4.5). A prevailing belief was that embracing sustainability would result in novel approaches enriched by a robust social orientation, without forfeiting technical expertise. Achieving this synthesis would necessitate the fusion of physical, human, and digital resources. Notably, the experts who shared insights during the course emphasized the essential interdisciplinary knowledge expected of sustainable managers (Fig. 6).

Furthermore, a theme that prominently emerged in the previous year retained its significance. Specifically, sustainable education continued to be heralded as the cornerstone for future civil society. Such education was thought to encompass not only academic lecturing but also hands-on immersion in actions to protect ecosystems across business and government domains. Each individual was perceived to possess a personal metric for gauging the sustainability of their actions, with priority assigned to the judicious and respectful use of available resources, placing the well-being of future generations over personal needs. This particular attribute garnered the highest score at the end of the course (4.7 vs. 4.2 at the beginning of the course). As shown in Fig. 6, 77% of students responded “very agree” at the end of the course, representing a notable increase from the 53% who responded likewise at the course outset.

The data presented in Fig. 6 raise important considerations regarding the extent to which students may contribute to driving change. When examining the results based on student type, it becomes evident that university students were thought to support change more significantly than high school students. This distinction is reflected in the delta of 0.9 (3.7 vs. 2.8), representing a discernible increase from the 0.7 delta (3.6 vs. 2.9) recorded in the previous year. Interestingly, the reason given by students was the same as that proposed by their counterparts in the previous year: not a lack of confidence in younger peers, but the recognition that it is difficult to identify concrete solutions to the sustainability challenge without possessing all the skills needed to address it effectively.

### Energy independence, sustainable certifications, and energy communities

**Figure 7:**
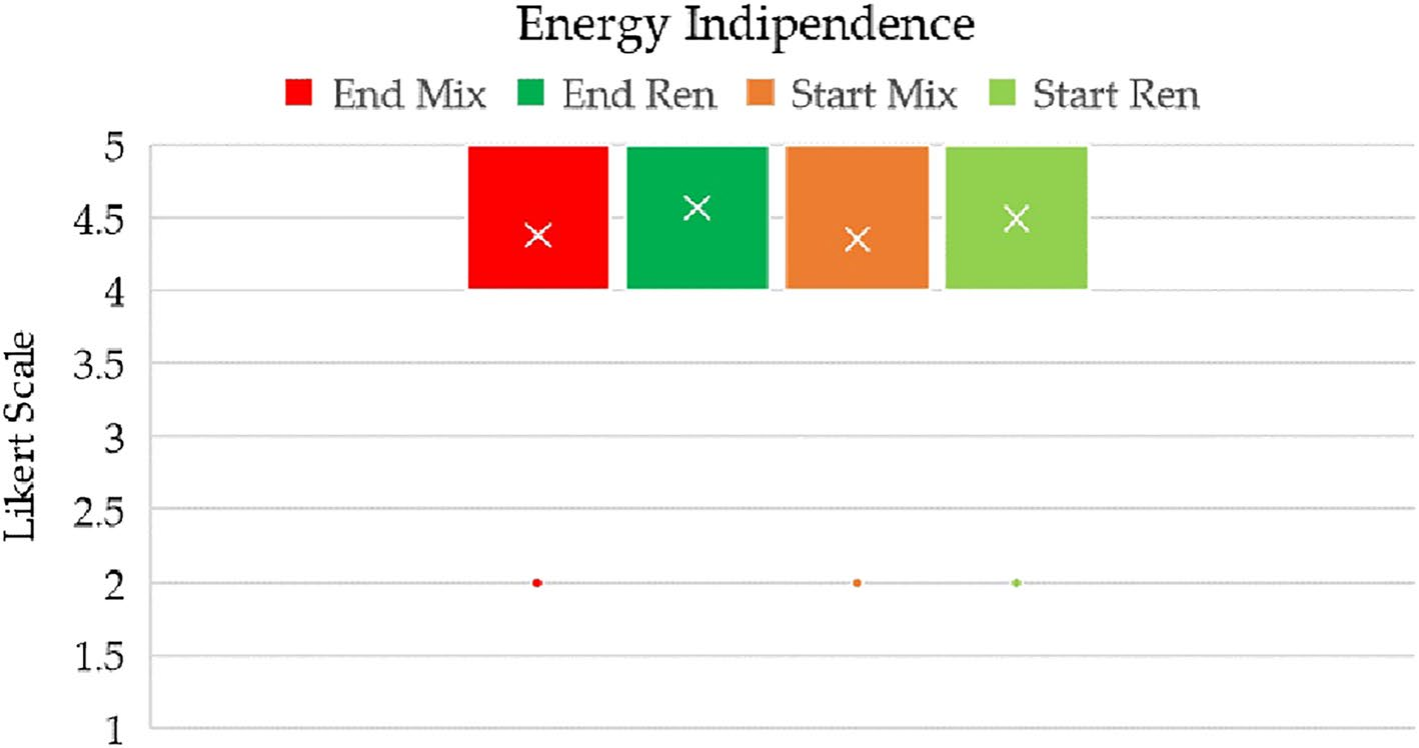
Energy independence achieved via a mix of domestic sources (Mix) or domestic renewable sources (Ren).

The issue of energy independence has become fundamental in countries that are highly reliant on imported energy sources. As previously described, this topic was explicitly introduced in the questionnaire. Notably, Italy has adopted policies that are progressively reducing its reliance on Russian gas (Fig. 7).

The outcome of this inquiry highlights the relevance of energy independence, which ranked second overall. An energy mix composed of renewable sources obtained an average value of 4.6 (+ 0.1 from the beginning to the end of the course). However, energy independence was still considered strategic even when the energy mix included fossil sources, as evidenced by the high rating of 4.4. Consequently, there existed a marginal gap of 0.2, stemming primarily from students’ economic perspectives.

The recent shock in energy costs has underscored the vulnerability of Italy’s economy to energy-related factors, with negative repercussions for both citizens and businesses. As a result, students were inclined towards managerial decisions that, while acknowledging the value of sustainability, also emphasized competitiveness and the avoidance of past errors.

**Figure 8:**
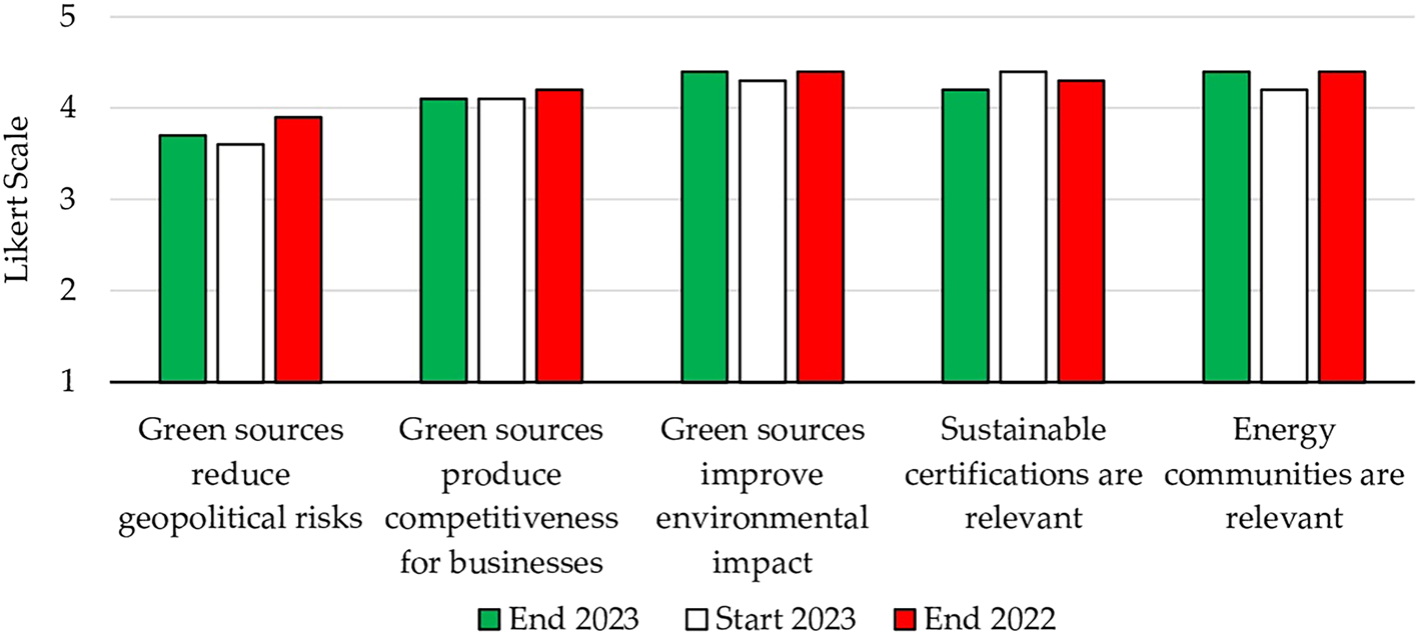
Impact of green sources, sustainable certifications, and energy communities.

The imperative of a sustainable shift is evident, as emphasized by the European Commission’s recognition of gas as a transitional resource towards a low-carbon society. For students, sourcing gas domestically was seen as more sustainable than importing gas, in alignment with the green transition. The favorable environmental impact and competitive advantage associated with green energy was further substantiated through students’ responses to other questionnaire items (Fig. 8)—see Supplementary material for the results of the Cronbach’s alpha.

In fact, students attributed a very high value to the notion that renewables contribute to an improved environmental impact (4.4). However, this value was somewhat tempered, largely owing to concerns about potential emission levels in biomass, which might exceed those of other renewable sources. Nevertheless, the environmental advantage over fossil fuels was evident to all. Competitive advantage also performed well, with a score of 4.1. This may be attributed to renewables’ potential to not only reduce business costs, but also enhance brand reputation by appealing to a growing segment of consumers increasingly focused on sustainability.

Surprisingly, the data related to geopolitical risks (3.7 vs. 3.6 at the beginning of the course, indicating a 0.2 decrease from the previous year) differed from the data on energy independence. Upon further investigation, it was discovered that students perceived these aspects in distinct ways. Energy sources may indeed be a potential cause of conflict, especially when these resources are crucial for the economy of the owning country. Moreover, geopolitical risks may be influenced by cultural aspects.

A declining trend was observed in the data concerning sustainable certifications. Although these certifications received a high value of 4.2, this represented a decrease of 0.1 from the previous year and 0.2 from the beginning of the course. This decline was attributed to a topic discussed in class, related to the Green Claims Directive. It seeks to ensure that consumers receive accurate and trustworthy information about the environmental attributes of the products they purchase.

Conversely, the topic of energy communities garnered positive feedback from students, earning a rating of 4.4 (up 0.2 from the beginning of the course). This highlights a strong curiosity about the concept and its potential long-term implications in reshaping social relations. Indeed, such communities are akin to timeshare investments. Additionally, attention was drawn to the need to implement energy communities even in large cities, and the idea that new professional roles are needed to facilitate these transformative shifts.

### Energy efficiency, energy habits, and renewable plant locations

**Figure 9:**
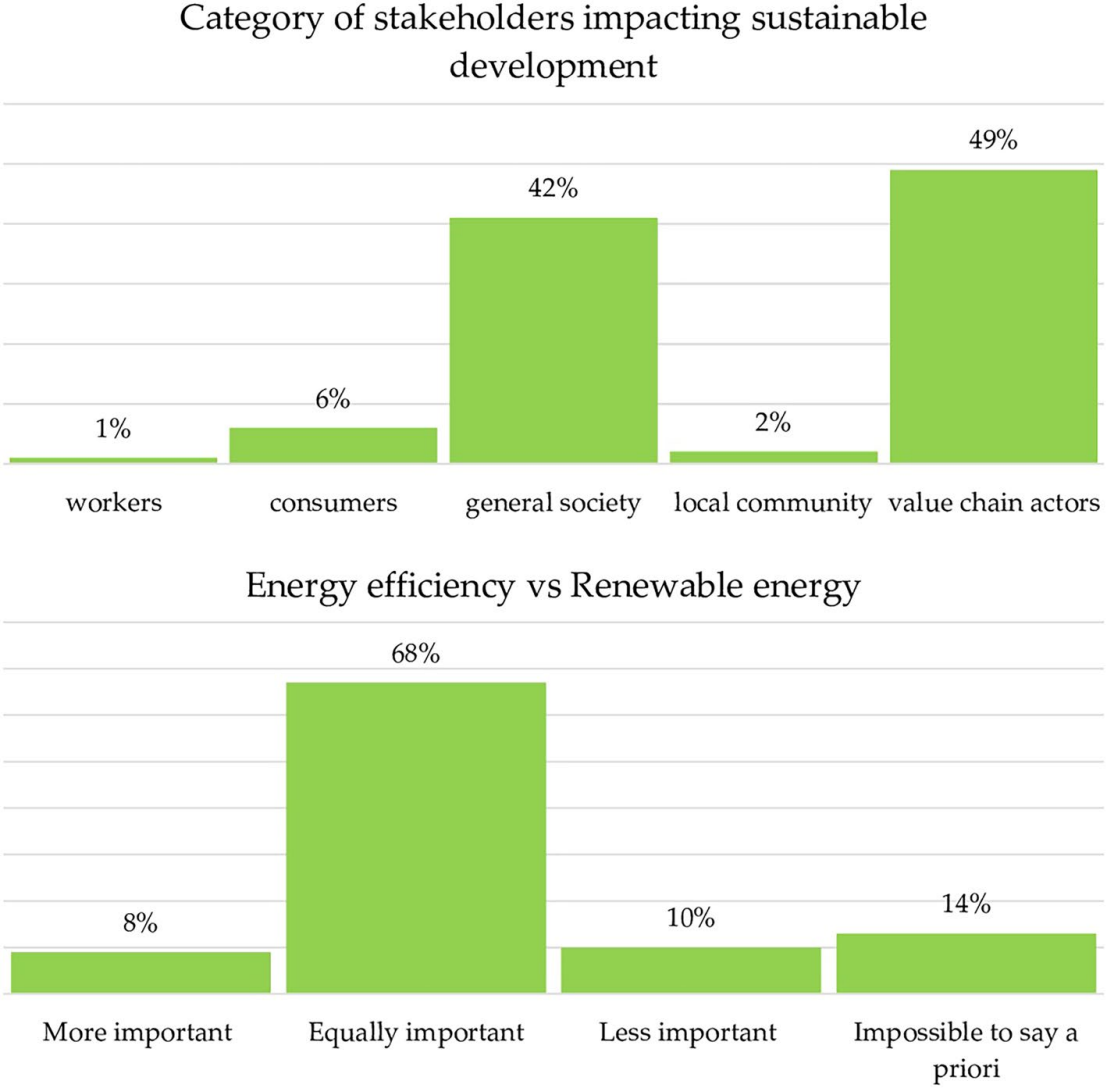
Stakeholder categories and the role of energy efficiency in the sustainable transition.

Sustainable change requires the active engagement of diverse stakeholder groups. The findings presented in Fig. 9 reveal that certain stakeholders were perceived to wield a more significant impact on final outcomes. Specifically, they show that business entities and participants in the general value chain (49%) and broader society (42%) were thought to hold the most impact. Interestingly, this result displays a reversal in the positions of these categories relative to the previous year (38% vs. 42%) and the course outset (41% vs. 45%). According to students, this shift could be attributed to businesses’ growing alignment with CSR principles, as well as their greater propensity to seek employment in environments where such principles are practiced.

Another crucial strategy for emissions reduction was thought to be energy efficiency interventions. While these are generally explored in much greater depth in non-management degree programs (due to their technical nature), students unequivocally emphasized that energy efficiency and renewable energy carry equal importance (68%). Notably, this value rose from the beginning of the course (60%) and was consistent with the previous year’s findings (65%).

Continuing along this trajectory, 42% of students deemed the competitiveness of emerging technologies as essential (representing a decrease of 10% from the beginning of the course). Conversely, the option of electrifying all uses was not considered strategically significant (7%). Nonetheless, students underlined the need for a shift in consumption behaviors (from 24% at the start of the course to 30% at the end of the course).

**Figure 10:**
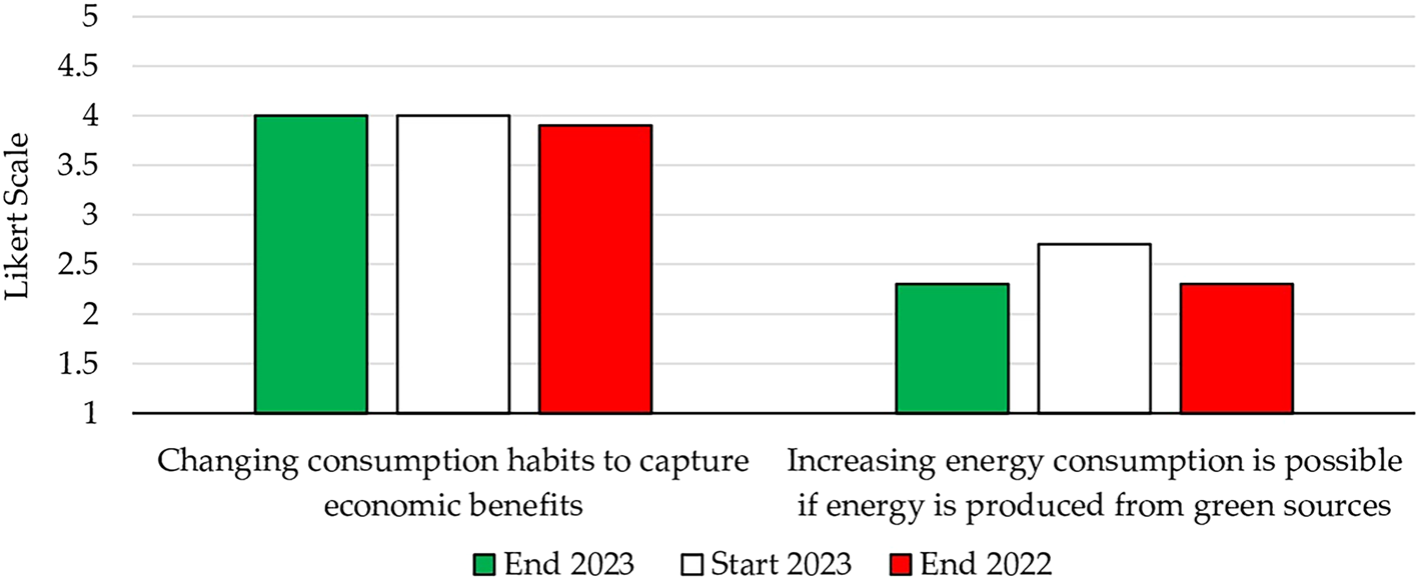
Energy habits related to green fuels.

Delving deeper into energy consumption habits, students expressed their willingness to modify these habits in order to capitalize on potential economic benefits (4.0). However, a concerning trend of unsustainable consumption behavior emerged, attributable to the concept of the green economy rebound (Fig. 10). Students were asked to reply to the following prompt: “I may even consume more because the environmental impact is reduced.” It is important to note that, while the use of green energy might align with this sentiment, it does not justify inappropriate use.

When comparing responses between the beginning and the end of the course, a shift was observed from 2.7 to 2.3, mirroring the situation in the previous year. Notably, a score of 2.3 indicated that students were more included to select a “little agree” response than a “not at all agree” response. Students recognized that this risk might be deemed acceptable, due to the perception that the use of renewable sources inherently contributes to environmental protection. However, students placed less emphasis on the specific consumption behaviors consumers should adopt, and this aspect was not consistently taken into account. Of note, for students, the rating “little agree” still carried a strongly negative implication.

**Figure 11:**
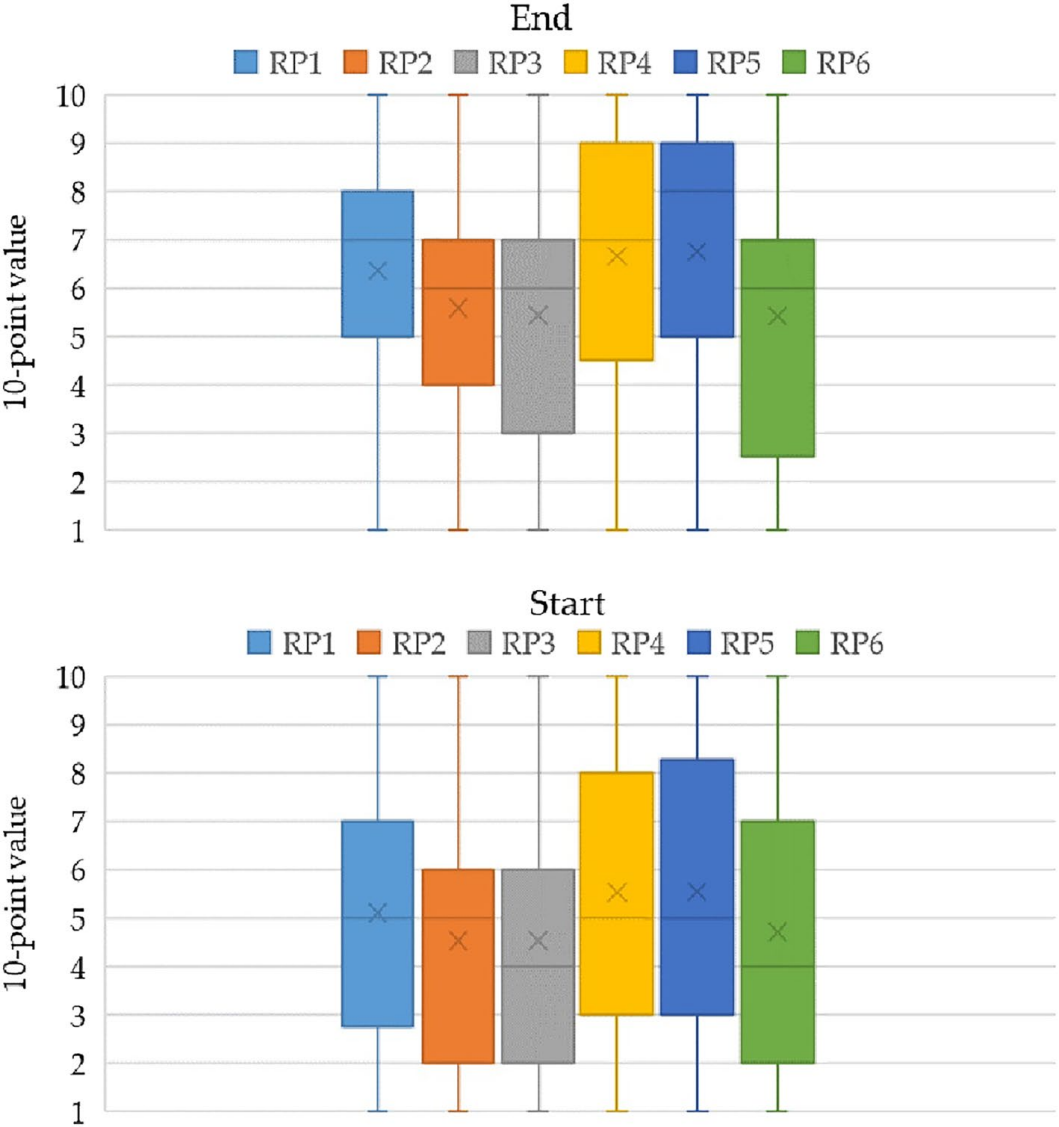
Location of renewable plants at the end and beginning of the course, respectively. RP1 = regardless of all factors (5.5, 4.7); RP2 = regardless of concerns from national politicians about losing electoral support (6.8, 5.5); RP3 = regardless of concerns from local politicians about losing electoral consensus (6.7, 5.5); RP4 = regardless of the specific installation site (e.g., near one’s residence, within one’s region of residence) (5.5, 4.5); RP5 = regardless of the type of substrate, considering its origin (e.g., local residue, extra-regional) (5.6, 4.5); RP6 = regardless of the energy source (e.g., solar, biomass) (6.4, 5.1).

The final section of the questionnaire aimed at exploring students' preferences, in the event that they came to be involved in a decision-making process to determine the location of a renewable energy plant. To capture a wider range of responses, this section employed a 10-point value scale (Fig. 11).

Based on the Kruskal–Wallis test (*χ*^2^ = 34.39, *p* < 0.000002), the differences between the mean ranks of certain groups were statistically significant. The post-hoc Dunn's test, employing a Bonferroni corrected alpha of 0.0033, indicated distinct mean rank differences among several pairs. The first three consisted of: RP2–RP4; RP2–RP3, and RP3–RP4. Conversely, the same was not verified in the data for the start of the course, for which the following values emerged (*χ*^2^ = 10.89, *p* = 0.054). The results show that the course exerted a positive influence, resulting in a relative increase across all questions, ranging from + 0.8 to + 1.3. Of notable significance were responses associated with challenging the “not in my term of office” (NIMTO) mindset, which achieved scores of 6.8 (for national politicians) and 6.7 (for local politicians). These scores underscore students’ inclination towards practical solutions for current issues. This suggests that electoral consensus must not only be cultivated within the present generation, but also valued by those to come. The guiding principle seems straightforward: adopt practical measures that genuinely enhance ecosystem equilibrium.

Another well-performing response pertained to the choice of renewable energy types, which scored 6.4. This aligns with the imperative of increasing Italy’s domestic resources. Among the various renewable options, environmental performance may vary, but the contribution remains vital. Students emphasized the need for future choices to emanate from transparent and collective initiatives.

Hence, the notion of unconditional acceptance of a renewable energy plant garnered a consensus score of 5.5. The same rating was assigned to scenarios in which the specific location was not considered. Of note, these ratings did not merely imply the presence of “not in my back yard” (NIMBY) syndrome. Rather, as students elaborated, the example cited often pertains to whether the resources generated arise from their actions or their potential waste. In the latter case, it was deemed sustainable to adopt behaviors that would mitigate any adverse impact. Furthermore, students emphasized that, while action needs to be taken, choices must be equitable and balanced. In particular, students found it inappropriate to transport certain wastes, and they also highlighted the lack of self-sufficiency in some areas. In this context, the question that probed students’ preferred geographical location for substrate use received a rating of 5.6.

### Multicriteria decision value

The final step of the questionnaire analysis involved aggregating all responses (Table 1). Table 1 summarizes the above findings, with two factors tending by approximation to the value of 5 (i.e., the maximum on the Likert scale): (i) sustainable education and (ii) energy independence (through renewable sources). Nevertheless, the subsequent items in the ranking highlight distinct political, strategic, and educational implications: (i) green subsidies; (ii) strategic independence, which remained significant even when reliant on fossil sources; (iii) the contribution of renewables to combating climate change; (iv) the pivotal role played by energy communities; and (v) the need for new professional roles (all with values that tend, by approximation, to 4.5).

**Table 1:** Ranking of factors pertinent to sustainable development in an engineering energy management course.

Acronym	Factors	Likert scale (1–5)	Sustainable index
*x*3	Sustainable education	4.7	X
*x*18	Energy independence (only renewable sources)	4.6	
*x*2	Relevance of subsidies (green sources)	4.5	X
*x*17	Energy independence (energy sources mix)	4.4	
*x*4	Green sources improve environmental impact	4.4	X
*x*1	Relevance of energy communities	4.4	X
*x*5	Need for new professional roles	4.3	X
*x*6	Relevance of sustainable certifications	4.2	X
*x*8	Green sources produce competitiveness for businesses	4.1	X
*x*9	Tax businesses not following sustainability	4.1	X
*x*11	Changing consumption habits to capture economic benefits	4.0	X
*x*13	Internet impacts sustainability	4.0	X
*x*15	Non-relevance of subsidies (fossil sources)	3.8	X
*x*10	Tax citizens not following sustainability	3.8	X
*x*16	Greenwashing does not support sustainability	3.8	X
*x*7	Green sources reduce geopolitical risks	3.7	X
*x*14	Increased energy consumption is possible if energy is produced from green sources	3.7	X
*x*19	University students can develop sustainable plans	3.7	
*x*12	Green energy self-production with no incentives	3.3	X
*x*20	High school students can develop sustainable plans	2.7	

At this point, it was possible to create a sustainable index, based on the questionnaire items, using MCDA. Sixteen factors were considered, as indicated by the “X” in the last column of Table 1. Several considerations guided the choice of factors: (i) only questions featuring a Likert scale were selected; (ii) only questions present in both questionnaire versions, enabling a comparison across years, were evaluated (thus, questions on energy independence were excluded); and (iii) questions regarding new generations were omitted, as they were perceived as more commendable proposals than executable actions. A high value on the sustainable index indicated strong performance. Of note, three factors (i.e., fossil source subsidies, increased energy consumption, greenwashing) deviated from this principle. Consequently, their reciprocals were calculated to render them comparable with the other 13 criteria.

**Figure 12:**
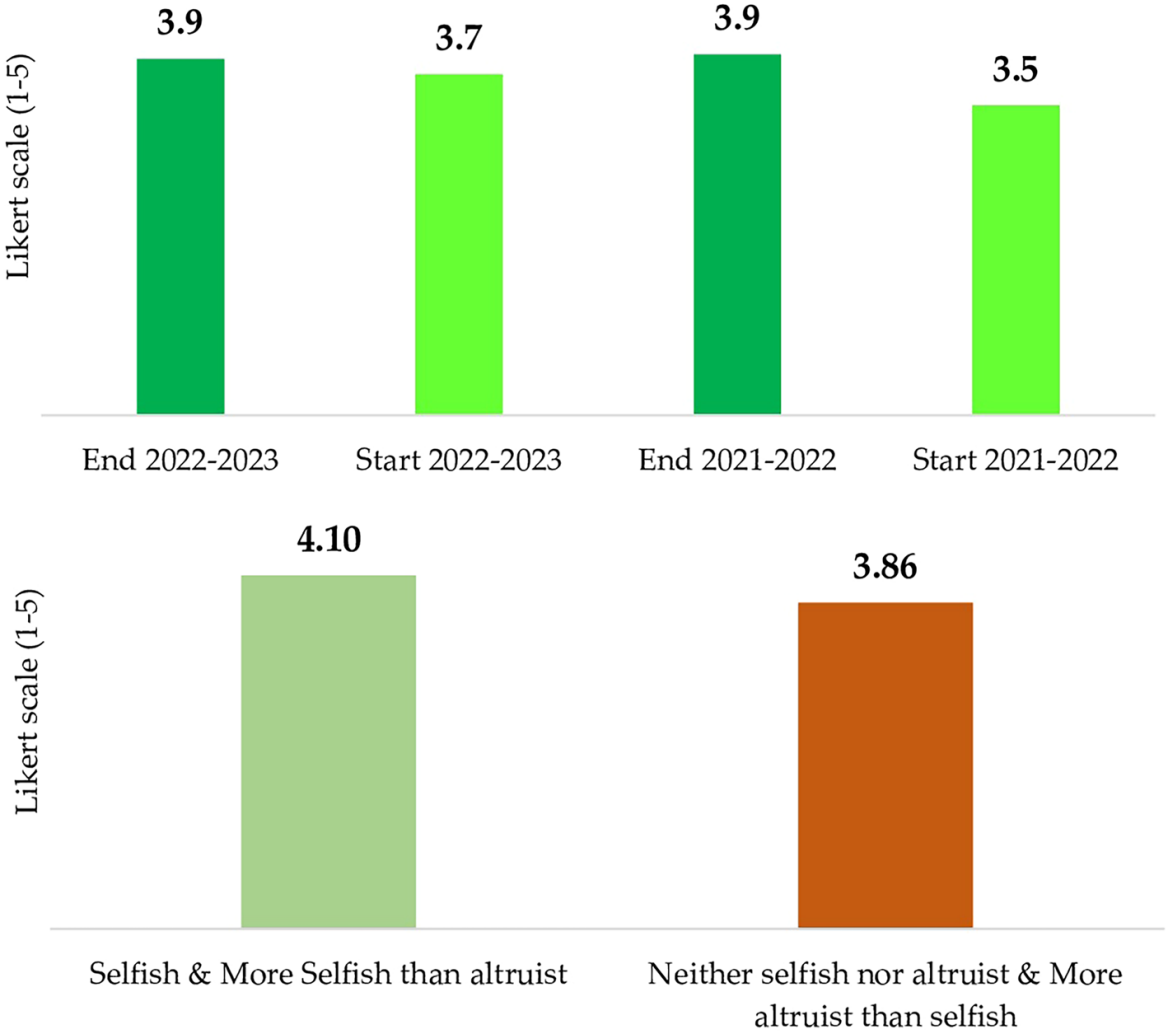
Sustainable index.

The results of the index objectively show that the course enhanced students’ sustainability performance across both years—Fig. 12. There was a discernible increase in students’ familiarity with sustainability topics compared to the previous year (3.7 vs. 3.5), and this trend culminated in a final value of 3.9, consistent with the previous year’s value. Moreover, aligning these findings with students’ individual characteristics, students with an altruistic disposition tended to be more supportive of this transformative shift (4.10 vs 3.86).

### Statistical measures

To lend greater significance to the results, a Kruskal Wallis test was once again conducted. At this stage, the analysis was specifically applied to two distinct contexts. The first pertained to the 16 criteria used for the sustainable index, wherein H_0_ was rejected (*χ*^2^ = 229.05, *p* < 0.001). This implied that the mean ranks of certain groups were indeed not equal. Subsequently, a post-hoc Dunn's test was employed with a Bonferroni corrected alpha of 0.00042. This test highlighted several differences in the mean ranks among the following pairs: *x*1– *x*7; *x*1– *x*10; *x*1– *x*11; *x*1– *x*12; *x*1– *x*14; *x*1– *x*15; *x*2– *x*7; *x*2– *x*10; *x*2– *x*11; *x*2– *x*12; *x*2– *x*13; *x*2– *x*14; *x*2– *x*15; *x*2– *x*16; *x*3– *x*5; *x*3– *x*6; *x*3– *x*7; *x*3– *x*8; *x*3– *x*9; *x*3–×10; *x*3– *x*11; *x*3– *x*12; *x*3– *x*13; *x*3– *x*14; *x*3– *x*15; *x*3– *x*16; *x*4– *x*7; *x*4– *x*10; *x*4– *x*11; *x*4– *x*12; *x*4– *x*14; *x*4– *x*15; *x*5– *x*7; *x*5– *x*10; *x*5–*x*12; *x*5– *x*14; *x*5– *x*15; *x*6– *x*7; *x*6– *x*12; *x*6– *x*15; *x*7– *x*9; *x*8– *x*12; *x*9–*x*12; *x*11– *x*12; *x*12– *x*13, and *x*12– *x*16.

The second context concerned responses that converged around an approximate value of 4.5, of which there were seven. The Kruskal–Wallis test showed that the differences in mean ranks for some groups was statistically significant (*χ*^*2*^ = 33.05, *p* < 0.001). Specifically, the post-hoc Dunn's test using a Bonferroni corrected alpha of 0.0024 indicated significant differences in the mean ranks of the following pairs:  *x*1*–x*3*;* *x*17– *x3*; *x3–x4*, and *x3–x5* (Table 2).

**Table 2:** Kruskal Wallis test—main ranking criteria.

Pair	*p*-value	Pair	*p*-value	Pair	*p*-value
*x*1*–* *x*2	0.173	*x*2–*x*18	0.398	*x*17–*x*5	0.554
*x*1*–* *x*17	0.897	*x*2–*x*3	< 0.009	*x*18–*x*3	0.074
*x*1*–* *x*18	0.027	*x2–x*4	0.222	*x*18–*x*4	0.039
*x*1*–* *x*3	< 0.00007	*x2–x*5	0.037	*x*18–*x*5	< 0.004
*x*1*–x*4	0.889	*x*17–*x*18	0.019	*x*3–*x*4	< 0.0002
*x*1*–x*5	0.471	*x*17–*x*3	< 0.00004	*x*3–*x*5	< 0.000003
*x*2–*x*17	0.136	*x*17–*x*4	0.778	*x*4–*x*5	0.389

In addition, a correlation matrix was employed to analyze the two distinct groups of criteria. In Table 3, the analysis related to the criteria comprising the sustainable index reveals that there were no notable high correlations suggesting non-random relationships. The highest correlations were observed for the following associations: 0.662 between potential taxes targeting those who do not follow sustainability principles among the stakeholder groups of businesses and citizens; 0.403 between the insignificance assigned to fossil fuel subsidies and the lack of support for sustainability in cases of greenwashing; and 0.389 between energy communities and sustainable certifications, as well as between the influence of green energy on competitiveness and its contribution to climate change.

**Table 3:** Correlation matrix referring to the 16 criteria of the sustainable index.

	*x*1	*x*2	*x*3	*x*4	*x*5	*x*6	*x*7	*x*8	*x*9	*x*10	*x*11	*x*12	*x*13	*x*14	*x*15	*x*16
*x*1	1															
*x*2	0.098	1														
*x*3	0.328	0.132	1													
*x*4	0.250	0.239	0.206	1												
*x*5	0.092	0.112	0.347	0.092	1											
*x*6	0.389	0.314	0.187	0.203	0.306	1										
*x*7	0.166	0.136	0.160	0.212	−0.039	0.062	1									
*x*8	0.262	0.131	0.247	0.389	0.174	0.231	0.241	1								
*x*9	0.299	0.123	0.231	0.103	0.088	0.094	0.106	0.225	1							
*x*10	0.186	0.026	0.211	0.110	0.102	0.138	0.046	0.207	0.662	1						
*x*11	0.254	−0.012	0.190	0.302	0.347	0.109	−0.170	0.243	0.279	0.321	1					
*x*12	−0.065	0.100	0.121	0.136	0.088	0.166	0.134	0.104	0.221	0.273	0.173	1				
*x*13	0.224	0.192	0.068	−0.022	0.178	0.214	0.200	0.286	0.059	−0.107	−0.019	0.115	1			
*x*14	0.166	0.092	0.158	−0.034	−0.099	−0.005	−0.140	0.031	0.070	0.031	0.193	−0.068	0.146	1		
*x*15	0.016	0.095	0.148	0.044	0.110	−0.067	0.022	0.106	−0.045	−0.078	−0.087	0.098	0.125	0.221	1	
*x*16	0.114	0.049	0.013	−0.066	−0.049	−0.010	−0.167	−0.050	0.002	−0.183	−0.019	−0.060	0.173	0.281	0.403	1

These results underscore the need for taxation strategies to encompass a comprehensive framework that involves all stakeholders. Additionally, they highlight the correlation between two attitudes contradictory to sustainable development: subsidies for environmentally impactful sources and deceptive claims of environmental initiatives by businesses that do not execute such projects. Furthermore, the concept of a community rests on a foundation of trust, which is as pivotal for nurturing energy communities as it is for developing credible sustainable labels. Finally, renewable energy effectively bridges economic and environmental dimensions.

In the context of the correlation matrix analysis for the first seven ranking criteria, differences emerged compared to the previous analysis, mainly due to the inclusion of the two criteria related to energy independence, although these criteria did exhibit significance. The highest correlation of 0.345 was recorded for sustainable education and the need for new job opportunities. This result underscores the imperative of establishing a positive feedback loop, connecting the realms of education and work.

### Financial-legal initiatives

During the experts' seminars, another need emerged, namely for technical profiles to be placed within the public administration, and in this regard it would be useful to contaminate their ideas with those of lawyers. In fact, changes that are also required in Europe in order to be ready for the ecological transition^67^. The transformation towards a lower-emission economy will require significant private and public investment. The financial sector will play an important role in financing global investment needs in the context of international climate policy and in directing capital flows towards sustainable investments. The decisive incentive for private investment is based on the return prospects. These are influenced in different ways by the effects of climate change and climate policy decisions such as the introduction of CO_2_ pricing. In addition, there may be information asymmetries that act as a hurdle for the sufficient mobilization of capital in sustainable projects, as they can stand in the way of the correct pricing of risks. The supply and demand for sustainable financial assets have increased significantly in recent years. Green bonds are bonds, whose proceeds are earmarked for the implementation of environmental and climate protection projects.

## Discussion

The literature highlights the potential for greater student engagement through the integration of the SDGs into teaching^68^, while also emphasizing the role of living labs in driving development^33^. In fact, the topic is very relevant^69^ because universities are responsible for sustainable development in communities^70^ and sustainable community engagement can foster the achievement of SDG 4 (Quality Education)^71^. The choice of the energy theme is considered fundamental to students' understanding of the role of this resource in global competitiveness^72^ and represents a fertile ground in which to combine interdisciplinary elements^53^. In addition, a concrete initiative could be to favour university housing in flats within energy communities^50^.

**Figure 13:**
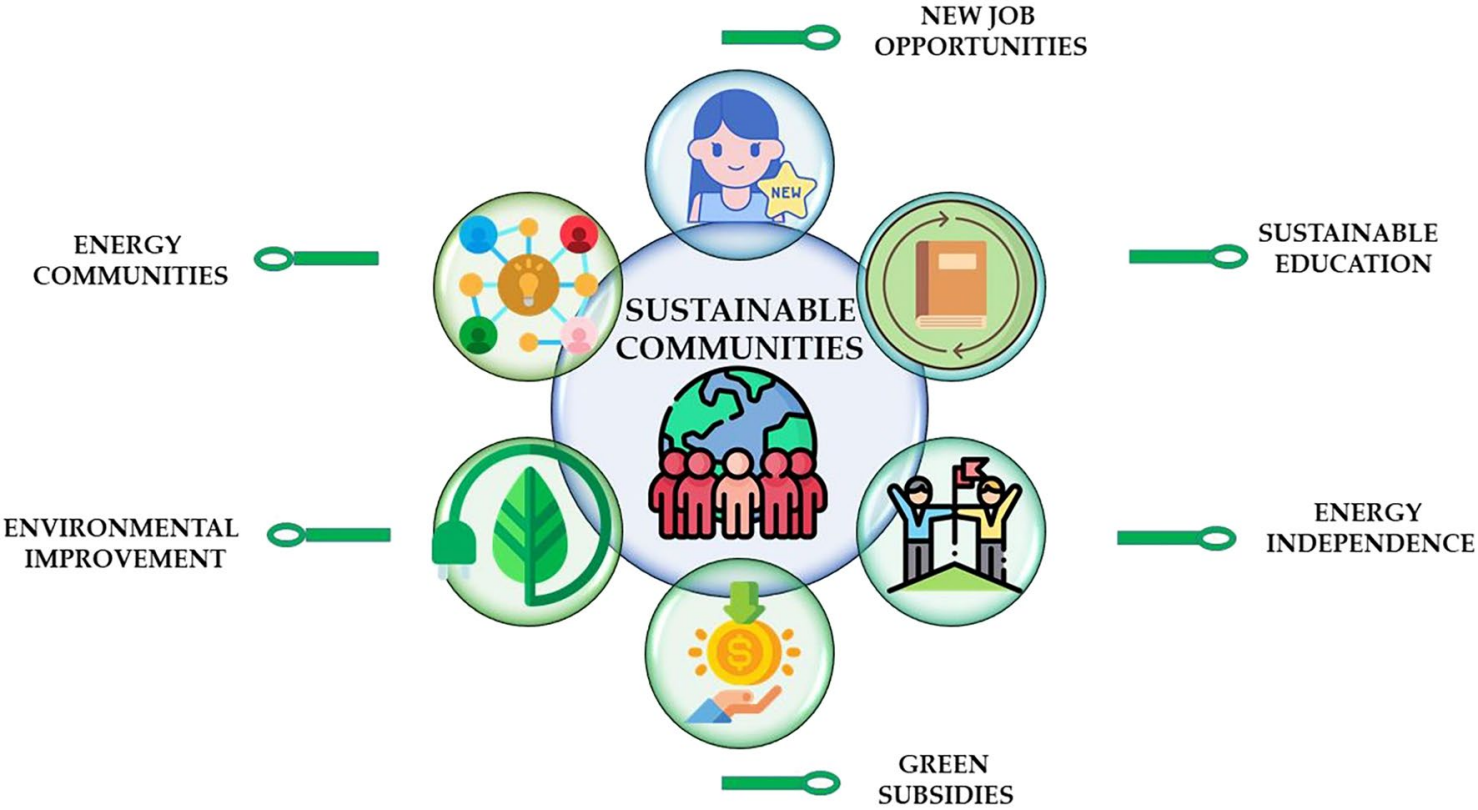
The role of sustainable communities in HEIs.

The present study aimed at providing a foundation for future research, based on the identification of key components of sustainable communities. In particular, the results referred to the energy context (Fig. 13).

Climate change is an indisputable reality, and renewable energy may play a pivotal role in countering this issue^73,74^. In this context, it is justifiable for policymakers to support and subsidize its development. Indeed, this strategy may be beneficial for all projects fostering the growth of the green economy. Similarly, the expansion of decentralized models demands the emergence of an increasing number of energy communities. However, the realization of these communities may encounter challenges, due to potential ideological conflicts among citizens and businesses. In this regard, the introduction of new professional roles could facilitate this transition.

Moreover, a pragmatic perspective prevails, placing significant emphasis on the concept of independence. It is crucial to note that this concept extends beyond the energy dimension. In fact, during the expert seminars, the necessity for attaining independence even from a material standpoint became evident. This implies two actions: (i) the identification, monitoring, and use of unused local raw materials; and ii) the promotion of recycling, recovery, and reuse practices to secure unavailable raw materials. This framework cannot be forward looking in the absence of sustainable education, which also emerged in the present analysis as the strategic foundation for the civil society of the future^53^. From this perspective, other implications come into view.

From a methodological perspective, the present study highlighted the advantage of employing diverse quantitative tools to compare a wealth of acquired data. Simultaneously, it highlighted the need to extend such analyses to encompass varying educational approaches. Indeed, the risk of “sustainable washing” was recognized as a phenomenon on par with greenwashing^15^.

From a managerial standpoint, four distinct characteristics come to the forefront, each serving as a resource bolstering the attainment of sustainable community objectives within the university context. First, the concept of interdisciplinary collaboration arises, due to the intricate nature of the significant climate shifts that have defined this century. The value of infusing academic discourse with insights from industry and public administration is apparent, given the complexity of these environmental changes. In this way, interdisciplinarity is able to support real problem solving and foster students' motivation and involvement in implementing the change required by sustainable development^75,76^.

Second, a pragmatic approach to sustainability emerges, centered on resolving challenges without causing harm to the majority of stakeholders. Unfortunately, adopting an ideological approach may lead to accepting certain choices without the support of empirical data and thorough analysis. Similarly, a lack of knowledge may result in decision-making inertia. Indeed, the literature has shown that educational institutions have not deeply embraced sustainability aspects in their curricula and in providing an appropriate learning environment^22^. A gear shift is needed, as the SDGs can help universities relate better to external stakeholders and society^68^.

The third facet involves fostering trust in the capabilities of younger individuals. An enduring sense of a sustainable community was evident when the previous year's students presented their projects, and this year’s students engaged by listening and interacting with their peers. This interaction could confirm existing beliefs or spur exploration of new alternatives, helping the students both pass exams and devise real-world solutions. Of note, the examination process involved the creation of final projects, characterized by frequent interim meetings (arranged at the student's discretion) with professors. This approach, albeit time-consuming, yielded two significant outcomes: (i) it captivated students' attention, fostering problem-solving skills and nurturing critical thinking aimed at continuous improvement; and (ii) a remarkable number of student projects evolved into enduring connections, resulting in long-term thesis work. Thus, for students to maintain ties with their university beyond their studies, it is imperative for universities to invest in human capital and fortify relationships with students. Thus, the teaching–learning environment has evolved and targeted and continuous efforts are needed for the transfer of skills^77^. This change requires the implementation of human resource management practices with socio-economic and psychological support within universities^78^.

Lastly, the fourth characteristic pertains to altruism. Striking a balance between personal gratification and organizational fulfilment is vital, as is the ability to navigate an external landscape that is in constant flux. Sustainability projects within universities foster civic and political involvement of students^79^ and the task of these institutions is to lead cultural change by listening students’ needs and passing their sense of responsibility to others^15^. Altruism encompasses not only human relationships, but also the ecosystem that sustains human existence.

## Conclusions

Sustainability is more than just a mere research topic; it signifies a novel approach to redefining the relationship between humanity and nature. It underscores that safeguarding the environment, alone, is insufficient. Rather, the pursuit of sustainability requires social and economic dimensions to be addressed, while transcending self-interest. Envisioning the future entails ensuring that forthcoming generations are afforded at least the same opportunities as the present one.

This transformative shift inevitably encompasses the realm of professional and personal training. Thus, integration of some of the SDGs into undergraduate courses is imperative, in alignment with their specific focal points. The present study quantified the impact of this integration on perceptions of sustainability issues among engineering students at an Italian university. Of note, the structure and content of the examination was strongly oriented toward sustainability.

The results affirm what the literature has already indicated: university courses have the capacity not only to increase students’ sustainable knowledge, but also to ignite profound curiosity among the new generation to explore these issues further. The fundamental premise of this approach revolves around two key elements. First, students create a self-selected project, which may be an individual endeavor or a group effort. This project must be geared towards resolving real-world issues, employing a quantitative approach. Second, students receive consistent guidance throughout the diverse phases of the project, facilitated by seminars led by experts and the preceding year's students.

Methodologically, the questionnaire can be replicated across other courses by incorporating specific items pertaining to the subjects taught. However, it is important to acknowledge the primary limitation of this study: alternative pedagogical approaches could yield more effective outcomes and consequently should be proposed and compared in terms of student satisfaction and the results achieved by the projects presented. Similarly, the sustainability index could be refined by including a broader array of criteria. This limitation adds to the problem that students do not always follow the lectures consistently and this could alter the final result and this aspect cannot be resolved in order to guarantee privacy when filling in the questionnaire. In addition, for future works it could be helpful to perform factor analysis and principal components analysis to investigate and reinforce the consistency of the questionnaire.

From an operational perspective, the present study introduced strategies for cultivating sustainable communities within HEIs, shedding light on the aspects that transform a university course into a hub for pragmatic ideas and projects. Specifically, six pillars (i.e., sustainable education, energy independence, green subsidies, environmental improvement, energy communities, professional opportunities) and four resources (i.e., interdisciplinary collaboration, pragmatism, confidence in youth competency, altruism) were identified. However, a notable limitation of the present approach is apparent: an expansion beyond the realm of energy could offer broader insights into sustainability. However, given the pervasive influence of energy topics across all sectors, it stands as a compelling and replicable case study.

Sustainability-focused courses present a significant opportunity that should extend even to individuals who have opted to discontinue formal education. Allocating European national funds in this direction would facilitate broader access. Crucially, participants would not merely be passive listeners, but actively engaged in project execution. University students themselves could serve as mentors, fostering a cross-pollination of ideas. In this way, a policy proposal for spending European funds emerges that is geared towards the involvement of university students in the training model as transmitters of knowledge to people who have stopped studying or who are interested in these issues. Further policy suggestions from this work are the strengthening of national independence towards which countries should strive with regard to energy and raw material components with the development of renewable energies and circular models. Such choices also require the provision of public funds directed only at projects that support pragmatic sustainability, which thus also allow for the development of the territory and does not undermine its independence at the onset of speculative phenomena or geopolitical risks.

In an era marked by rapid digitization, it remains paramount to recognize that education provides the precious gift of time, safeguarding the interests of both current and future students. Their deepened understanding of pertinent issues, coupled with heightened sensitivity, may pave the way for achieving something difficult yet profoundly beautiful. Just as sowing seeds demands patience to witness of blooming flowers in nature, our actions and choices must be patient investments in rendering environment more habitable for all. This objective finds its realization through the cultivation of sustainable communities.

### Ethics statement

Given that the research is a non-experimental voluntary survey, no ethical approval is necessary^53^. Indeed, the survey’s scope and objective were defined in such a way that the information collected via the questionnaire did not contain any sensitive data, minimized the processing of users’ personal data, and was gathered in a way that the data subjects are not identifiable under any circumstances ^80^. Furthermore, the self-administered survey that is non-experimental in nature was conducted under complete anonymity for the participants, following the legal duty of General Data Protection Regulation (GDPR) (EU) 2016/679. No personal or sensitive information that can be used to identify the respondents were collected. Besides, the consent of the respondents to partake in the online survey were seek before the survey was executed by including an electronic informed consent in the online survey form. All procedures were performed in accordance with relevant guidelines. The current Italian legislation does not require ethical approval for surveys involving humans related to this type of analysis.

### Supplementary Information

Supplementary Information.

## Data Availability

All data generated or analyzed during the present study are included in this article (and its supplementary information files).
