# Supplementary material for: Strategies for developing sustainable communities in higher education institutions
Source: Sci Rep. 2023 Nov 23;13:20596. doi: 10.1038/s41598-023-48021-8 (PMC10667548; doi:10.1038/s41598-023-48021-8)
Supplement: Supplementary file 1 — Supplementary Information. [file 41598_2023_48021_MOESM1_ESM.docx]

**Supplementary material**

**Questionnaire. All data in units.**

| **1** | **Indicate your A.Y. in which you started your master's degree** | END | | START | |  |
| --- | --- | --- | --- | --- | --- | --- |
|  | 2020-2021 | | 1 | | 2 | |
|  | 2021-2022 | | 26 | | 27 | |
|  | 2022-2023 | | 72 | | 65 | |
| **2** | **How old are you** | | END | | START | |
|  | 22 | | 24 | | 25 | |
|  | 23 | | 36 | | 32 | |
|  | 24 | | 19 | | 14 | |
|  | 25 | | 7 | | 12 | |
|  | 26 | | 7 | | 7 | |
|  | 27 | | 6 | | 2 | |
|  | 28 | | 0 | | 2 | |
|  | 29 | | 0 | | 0 | |
| **3** | **Indicate your gender** | | END | | START | |
|  | Man | | 65 | | 58 | |
|  | Woman | | 34 | | 35 | |
|  | I prefer not to answer | | 0 | | 1 | |
| **4** | **In which area of Italy did you study in high school?** | | END | | START | |
|  | Northern Italy | | 2 | | 1 | |
|  | Central Italy | | 83 | | 80 | |
|  | Southern Italy | | 14 | | 13 | |
|  | Other [More] | | 0 | | 0 | |
| **5** | **Indicate your occupation** | | END | | START | |
|  | Student | | 60 | | 65 | |
|  | Student and worker | | 23 | | 15 | |
|  | Student and part-time worker (seasonal) | | 16 | | 14 | |
| **6** | **Indicate who you live with** | | END | | START | |
|  | With your family | | 78 | | 75 | |
|  | With roommates | | 19 | | 17 | |
|  | With your partner(s) | | 0 | | 0 | |
|  | On my own | | 2 | | 2 | |
| **7** | **How do you define your character?** | | END | | START | |
|  | Selfish | | 0 | | 0 | |
|  | More selfish than altruistic | | 3 | | 6 | |
|  | Neither selfish nor altruistic | | 14 | | 11 | |
|  | More altruistic than selfish | | 69 | | 51 | |
|  | Altruistic | | 13 | | 26 | |
| **8** | **Are you thinking more about the present or the future?** | | END | | START | |
|  | Present | | 16 | | 16 | |
|  | Future | | 65 | | 58 | |
|  | Undecided | | 18 | | 20 | |

| **9** | **What does sustainability concern?** | END | START |
| --- | --- | --- | --- |
|  | Environmental aspects | 1 | 6 |
|  | Economic aspects | 1 | 1 |
|  | Social aspects | 0 | 0 |
|  | A mix of social, economic and environmental aspects | 97 | 87 |
| **10** | **How often do you spend time as a volunteer?** | END | START |
|  | Never | 35 | 35 |
|  | Rarely | 31 | 33 |
|  | Sometimes | 20 | 16 |
|  | Often | 10 | 7 |
|  | Always | 3 | 3 |
| **11** | **How often do you make recycling collection?** | END | START |
|  | Never | 0 | 1 |
|  | Rarely | 3 | 3 |
|  | Sometimes | 5 | 9 |
|  | Often | 35 | 19 |
|  | Always | 56 | 62 |
| **12** | **How often do you do practice sport?** | END | START |
|  | Never | 1 | 1 |
|  | Rarely | 16 | 14 |
|  | Sometimes | 17 | 16 |
|  | Often | 38 | 45 |
|  | Always | 27 | 18 |
| **13** | **How often do you buy sustainable products?** | END | START |
|  | Never | 0 | 1 |
|  | Rarely | 9 | 11 |
|  | Sometimes | 55 | 49 |
|  | Often | 34 | 32 |
|  | Always | 1 | 1 |
| **14** | **How often do you opt to make green choices with transportation?** | END | START |
|  | Never | 6 | 5 |
|  | Rarely | 19 | 18 |
|  | Sometimes | 29 | 29 |
|  | Often | 33 | 31 |
|  | Always | 12 | 11 |
| **15** | **How often do you need to be in touch with nature?** | END | START |
|  | Never | 1 | 1 |
|  | Rarely | 4 | 3 |
|  | Sometimes | 34 | 39 |
|  | Often | 47 | 46 |
|  | Always | 13 | 5 |
| **16** | **How many hours do you walk on average in a day (no weekends)?** | END | START |
|  | less than one km | 5 | 6 |
|  | between one and three kilometers | 48 | 39 |
|  | between three and six kilometers | 38 | 40 |
|  | more than six kilometers | 8 | 9 |

| **17** | **Willingness to pay (WTP) for a kWh produced from fossil sources (cent€/kWh)?** | END | START |
| --- | --- | --- | --- |
|  | 5 | 15 | 13 |
|  | 10 | 19 | 12 |
|  | 15 | 25 | 18 |
|  | 20 | 15 | 19 |
|  | 25 | 6 | 19 |
|  | 30 | 12 | 7 |
|  | 35 | 7 | 6 |
|  | 40 | 0 | 0 |
|  | 45 | 0 | 0 |
|  | 50 | 0 | 0 |
| **18** | **WTP for a kWh produced from renewables (cent€/kWh)?** | END | START |
|  | 5 | 2 | 4 |
|  | 10 | 7 | 6 |
|  | 15 | 17 | 15 |
|  | 20 | 16 | 15 |
|  | 25 | 17 | 18 |
|  | 30 | 20 | 15 |
|  | 35 | 11 | 9 |
|  | 40 | 5 | 11 |
|  | 45 | 3 | 0 |
|  | 50 | 1 | 1 |
| **19** | **Willingness to sell (WTS) for a kWh produced from fossil sources (cent€/kWh)?** | END | START |
|  | 5 | 10 | 11 |
|  | 10 | 15 | 8 |
|  | 15 | 28 | 18 |
|  | 20 | 16 | 14 |
|  | 25 | 7 | 18 |
|  | 30 | 9 | 13 |
|  | 35 | 8 | 4 |
|  | 40 | 3 | 2 |
|  | 45 | 0 | 3 |
|  | 50 | 3 | 3 |
| **20** | **WTS for a kWh produced from fossil sources (cent€/kWh)?** | END | START |
|  | 5 | 1 | 6 |
|  | 10 | 12 | 13 |
|  | 15 | 13 | 4 |
|  | 20 | 26 | 14 |
|  | 25 | 17 | 18 |
|  | 30 | 9 | 16 |
|  | 35 | 8 | 14 |
|  | 40 | 9 | 8 |
|  | 45 | 4 | 0 |
|  | 50 | 0 | 1 |

| **21** | **Energy communities are important. Do you share this view?** | END | START |
| --- | --- | --- | --- |
|  | strongly disagree | 0 | 0 |
|  | disagree | 1 | 2 |
|  | undecided | 5 | 10 |
|  | agree | 48 | 50 |
|  | strongly agree | 45 | 32 |
| **22** | **Sustainable certifications are important. Do you share this view?** | END | START |
|  | strongly disagree | 0 | 0 |
|  | disagree | 4 | 0 |
|  | undecided | 7 | 9 |
|  | agree | 48 | 43 |
|  | strongly agree | 38 | 42 |
| **23** | **Subsidies for the production of green sources are important. Do you share this view?** | END | START |
|  | strongly disagree | 0 | 0 |
|  | disagree | 0 | 1 |
|  | undecided | 3 | 3 |
|  | agree | 42 | 32 |
|  | strongly agree | 54 | 58 |
| **24** | **Fossil fuel subsidies are important. Do you share this view?** | END | START |
|  | strongly disagree | 28 | 15 |
|  | disagree | 34 | 29 |
|  | undecided | 23 | 27 |
|  | agree | 8 | 18 |
|  | strongly agree | 6 | 5 |
| **25** | **What value do you recognize to the subsidy for green energy produced and self-consumed (cent€/kWh)?** | END | START |
|  | 0 | 0 | 0 |
|  | 1 | 0 | 0 |
|  | 2 | 1 | 0 |
|  | 3 | 1 | 2 |
|  | 4 | 2 | 6 |
|  | 5 | 12 | 19 |
|  | 6 | 11 | 2 |
|  | 7 | 24 | 16 |
|  | 8 | 28 | 19 |
|  | 9 | 9 | 11 |
|  | 10 | 11 | 19 |

| **26** | **What value do you recognize to the subsidy for fossil energy produced and self-consumed (cent€/kWh)?** | END | START |
| --- | --- | --- | --- |
|  | 0 | 13 | 14 |
|  | 1 | 9 | 4 |
|  | 2 | 17 | 9 |
|  | 3 | 12 | 10 |
|  | 4 | 12 | 12 |
|  | 5 | 19 | 20 |
|  | 6 | 6 | 5 |
|  | 7 | 6 | 11 |
|  | 8 | 3 | 6 |
|  | 9 | 2 | 0 |
|  | 10 | 0 | 3 |
| **27** | **Green sources reduce geopolitical risks. Do you share this view?** | END | START |
|  | strongly disagree | 1 | 3 |
|  | disagree | 9 | 4 |
|  | undecided | 32 | 30 |
|  | agree | 37 | 43 |
|  | strongly agree | 20 | 14 |
| **28** | **Green sources produce competitive advantage for enterprises. Do you share this view?** | END | START |
|  | strongly disagree | 1 | 0 |
|  | disagree | 1 | 1 |
|  | undecided | 12 | 16 |
|  | agree | 55 | 51 |
|  | strongly agree | 30 | 26 |
| **29** | **Energy independence (obtained from a mix of sources produced in one's own national territory) results in an advantage for the country.** | END | START |
|  | strongly disagree | 0 | 0 |
|  | disagree | 1 | 1 |
|  | undecided | 5 | 7 |
|  | agree | 49 | 44 |
|  | strongly agree | 44 | 42 |
| **30** | **Energy independence (obtained only from renewable sources in one's own national territory) results in an advantage for the country.** | END | START |
|  | strongly disagree | 0 | 0 |
|  | disagree | 0 | 1 |
|  | undecided | 6 | 7 |
|  | agree | 31 | 31 |
|  | strongly agree | 62 | 55 |

| **31** | **Are you in favor of penalties/taxes for companies that do not follow sustainability principles?** | END | START |
| --- | --- | --- | --- |
|  | strongly disagree | 2 | 1 |
|  | disagree | 2 | 3 |
|  | undecided | 11 | 10 |
|  | agree | 47 | 51 |
|  | strongly agree | 37 | 29 |
| **32** | **Are you in favor of penalties/taxes for citizens who do not follow principles of sustainability?** | END | START |
|  | strongly disagree | 2 | 0 |
|  | disagree | 5 | 8 |
|  | undecided | 20 | 21 |
|  | agree | 52 | 44 |
|  | strongly agree | 20 | 21 |
| **33** | **Sustainable education is a pillar of civil society. Do you share this view?** | END | START |
|  | strongly disagree | 0 | 0 |
|  | disagree | 2 | 1 |
|  | undecided | 2 | 2 |
|  | agree | 20 | 41 |
|  | strongly agree | 75 | 50 |
| **34** | **Green sources reduce environmental impact. Do you share this opinion?** | END | START |
|  | strongly disagree | 0 | 0 |
|  | disagree | 1 | 2 |
|  | undecided | 11 | 12 |
|  | agree | 38 | 40 |
|  | strongly agree | 49 | 40 |
| **35** | **The development of new professionals is necessary for the ecological transition. Do you share this view?** | END | START |
|  | strongly disagree | 0 | 0 |
|  | disagree | 3 | 1 |
|  | undecided | 8 | 5 |
|  | agree | 46 | 55 |
|  | strongly agree | 42 | 33 |
| **36** | **I use green sources and no longer fossil sources, so I modify my consumption habits to take advantage of the potential economic benefits. Do you share this view?** | END | START |
|  | strongly disagree | 1 | 1 |
|  | disagree | 2 | 2 |
|  | undecided | 14 | 14 |
|  | agree | 64 | 57 |
|  | strongly agree | 18 | 20 |

| **37** | **I use green sources and no longer fossil sources, so I can also consume more because the unit environmental impact is reduced. Do you share this view?** | END | START |
| --- | --- | --- | --- |
|  | strongly disagree | 26 | 8 |
|  | disagree | 35 | 37 |
|  | undecided | 18 | 29 |
|  | agree | 18 | 16 |
|  | strongly agree | 2 | 4 |
| **38** | **The younger generation (students in university) are able to implement a sustainable development plan in practice. Do you share this opinion?** | END | START |
|  | strongly disagree | 2 | 1 |
|  | disagree | 3 | 5 |
|  | undecided | 25 | 30 |
|  | agree | 57 | 46 |
|  | strongly agree | 12 | 12 |
| **39** | **New generations (students in high school) are able to implement a sustainable development plan in practice. Do you share this opinion?** | END | START |
|  | strongly disagree | 11 | 6 |
|  | disagree | 32 | 22 |
|  | undecided | 29 | 30 |
|  | agree | 24 | 30 |
|  | strongly agree | 3 | 6 |
| **40** | **Internet use affects sustainability. Do you share this view?** | END | START |
|  | strongly disagree | 0 | 1 |
|  | disagree | 2 | 3 |
|  | undecided | 17 | 20 |
|  | agree | 56 | 52 |
|  | strongly agree | 24 | 18 |
| **41** | **Greenwashing helps sustainable development. Do you share this opinion?** | END | START |
|  | strongly disagree | 39 | 17 |
|  | disagree | 25 | 13 |
|  | undecided | 13 | 35 |
|  | agree | 20 | 26 |
|  | strongly agree | 2 | 3 |
| **42** | **I am willing to self-produce green energy at my home even without receiving incentives, that is, neither explicitly (through incentives) nor implicitly (through exemptions, tax deductions, etc.) How much do you agree with this statement?** | END | START |
|  | strongly disagree | 5 | 1 |
|  | disagree | 14 | 17 |
|  | undecided | 31 | 28 |
|  | agree | 40 | 45 |
|  | strongly agree | 9 | 3 |
| **43** | **For the purpose of achieving climate neutrality, how important do you think it is to use methodologies that increase energy efficiency with respect to the development of energy production from renewable sources ?** | END | START |
|  | More important | 9 | 9 |
|  | Equally important | 67 | 56 |
|  | Less important | 10 | 14 |
|  | Impossible to say a priori | 13 | 15 |
| **44** | **Do you think the decarbonization of a system like Italy's is achievable through the development and application of new technologies?** | END | START |
|  | Yes | 8 | 6 |
|  | Yes, as long as all possible uses are electrified | 7 | 5 |
|  | Yes, as long as technologies currently under development are also made competitive, in addition to the massive use of electricity | 42 | 49 |
|  | No, a change in our behaviors is also necessary | 30 | 23 |
|  | I don't know. | 12 | 11 |
| **45a** | **Most suitable approach through implementation of renewable plants regardless of type (e.g., solar,bioma** | END | START |
|  | 1 | 2 | 4 |
|  | 2 | 11 | 19 |
|  | 3 | 3 | 10 |
|  | 4 | 4 | 10 |
|  | 5 | 7 | 10 |
|  | 6 | 16 | 12 |
|  | 7 | 17 | 7 |
|  | 8 | 25 | 9 |
|  | 9 | 8 | 5 |
|  | 10 | 6 | 8 |
| **45b** | **Most suitable approach through the implementation of renewable plants regardless of the substrate considered (e.g.**  **Local residues, extra-regional residues)** | END | START |
|  | 1 | 2 | 7 |
|  | 2 | 12 | 21 |
|  | 3 | 5 | 6 |
|  | 4 | 11 | 12 |
|  | 5 | 14 | 17 |
|  | 6 | 16 | 12 |
|  | 7 | 19 | 8 |
|  | 8 | 13 | 4 |
|  | 9 | 4 | 5 |
|  | 10 | 3 | 2 |

| **45c** | **More suitable approach through the implementation of renewable plants regardless of the place of installation (e.g.near home, in our region)** | END | START |
| --- | --- | --- | --- |
|  | 1 | 5 | 10 |
|  | 2 | 11 | 19 |
|  | 3 | 9 | 7 |
|  | 4 | 7 | 12 |
|  | 5 | 13 | 16 |
|  | 6 | 15 | 10 |
|  | 7 | 22 | 6 |
|  | 8 | 8 | 4 |
|  | 9 | 3 | 6 |
|  | 10 | 6 | 4 |
| **45d** | **More suitable approach through the implementation of renewable plants regardless of the local politician's fear of losing electoral consensus** | END | START |
|  | 1 | 3 | 10 |
|  | 2 | 9 | 11 |
|  | 3 | 8 | 13 |
|  | 4 | 4 | 7 |
|  | 5 | 5 | 7 |
|  | 6 | 8 | 7 |
|  | 7 | 12 | 7 |
|  | 8 | 22 | 10 |
|  | 9 | 12 | 6 |
|  | 10 | 16 | 16 |
| **45e** | **More suitable approach through the implementation of renewable plants regardless of the national politician's fear of losing electoral consensus** | END | START |
|  | 1 | 1 | 9 |
|  | 2 | 10 | 11 |
|  | 3 | 8 | 14 |
|  | 4 | 4 | 7 |
|  | 5 | 5 | 8 |
|  | 6 | 9 | 7 |
|  | 7 | 11 | 5 |
|  | 8 | 23 | 10 |
|  | 9 | 11 | 8 |
|  | 10 | 17 | 15 |

| **45f** | **Most suitable approach through the implementation of renewable facilities regardless of all** | END | START |
| --- | --- | --- | --- |
|  | 1 | 9 | 12 |
|  | 2 | 15 | 19 |
|  | 3 | 2 | 11 |
|  | 4 | 7 | 6 |
|  | 5 | 14 | 10 |
|  | 6 | 11 | 5 |
|  | 7 | 17 | 11 |
|  | 8 | 11 | 9 |
|  | 9 | 4 | 4 |
|  | 10 | 9 | 7 |
| **46** | **Which one, among these stakeholder categories, does most influence sustainable development?** | END | START |
|  | workers | 1 | 1 |
|  | consumers | 6 | 11 |
|  | general society | 41 | 42 |
|  | local community | 2 | 1 |
|  | enterprises (value chain actors) | 49 | 39 |

Table A1. Summary statistics of the variables considering the answers provided at the end of the course. Column (4) reports the p-value of the Shapiro-Wilk test for normality distribution. Column (5) reports the value of the Cronbach’s alpha for the items analyzed jointly.

|  | **(1)** | **(2)** | **(3)** | **(4)** | **(5)** |
| --- | --- | --- | --- | --- | --- |
|  | **N** | **Mean** | **SD** | **Shapiro-Wilk (p-value)** | **Cronbach’s alpha** |
| Volunteering | 99 | 2.15 | 1.11 | <0.01 | 0.415 |
| Separate collection | 99 | 4.45 | 0.73 | <0.01 |  |
| Sport | 99 | 3.74 | 1.06 | 0.05 |  |
| Sustainable shopping | 99 | 3.27 | 0.64 | 0.04 |  |
| Sustainable mobility | 99 | 3.26 | 1.09 | 0.94 |  |
| Nature | 99 | 3.67 | 0.79 | 0.07 |  |
| Relevance of subsidies (green sources) | 99 | 4.52 | 0.56 | <0.01 | 0.408 |
| Relevance of subsidies (fossil fuel) | 99 | 2.29 | 1.15 |  |  |
| Green energy self-production with no incentives | 99 | 3.47 | 1.01 |  |  |
| Tax citizens not following sustainability | 99 | 3.84 | 0.88 | <0.01 |  |
| Tax businesses not following sustainability | 99 | 4.16 | 0.85 | <0.01 |  |
| Green sources reduce geopolitical risks | 99 | 3.67 | 0.94 | 0.243 | 0.595 |
| Green sources improve environmental impact | 99 | 4.36 | 0.72 | <0.01 |  |
| Relevance of energy communities | 99 | 4.38 | 0.63 | <0.01 |  |
| Green sources produce competitiveness for businesses | 99 | 4.13 | 0.74 | <0.01 |  |
| Relevance of sustainable certifications | 99 | 4.25 | 0.76 | <0.01 |  |
| Sustainable education | 99 | 4.70 | 0.61 | <0.01 |  |
| Energy independence (only renewable sources) | 99 | 4.57 | 0.61 | <0.01 |  |
| Energy independence (energy sources mix) | 99 | 4.37 | 0.63 | <0.01 |  |
| Need for new professional roles | 99 | 4.28 | 0.74 | <0.01 |  |
| Changing consumption habits to capture economic benefits | 99 | 3.97 | 0.71 | <0.01 |  |
| Internet impacts sustainability | 99 | 4.03 | 0.71 | <0.01 |  |
| Greenwashing does not support sustainability | 99 | 3.80 | 1.22 | <0.01 |  |
| Increased energy consumption is possible if energy is produced from green sources | 99 | 2.26 | 1.07 | 0.467 |  |
| University students can develop sustainable plans | 99 | 3.75 | 0.79 | <0.01 |  |
| High school students can develop sustainable plans | 99 | 2.76 | 1.04 | 0.243 |  |

For the interpretation of the Shapiro-Wilk test for normality, if the chosen alpha level is 0.05 as is the case here and the p-value is less than 0.05, and then the null hypothesis that the data are normally distributed is rejected. As we can see from Table A1 in Appendix A1, for most variables, the normality assumption is rejected and so our choice of using a nonparametric Kruskal-Wallis test is fully justified.

Cronbach’s alpha is a measure of internal consistency, that is, how closely related a set of items are as a group. It is considered a measure of scale reliability. Cronbach’s alpha ranges from 0 to 1. A value of zero indicates that there is no correlation between the items while a value of one indicates that they are perfectly correlated. What is an acceptable range for Cronbach’s alpha to ensure internal consistency changes according to the domain of application. Given that a high Cronbach’s alpha is a necessary condition for reliability, but it’s not sufficient for determining validity it could be useful for further developments to use factor analysis and principal components analysis to reinforce the consistency of the items of the questionnaire..
